# Introduction and methods of the evidence-based guidelines for the diagnosis and management of autism spectrum disorder by the Italian National Institute of Health

**DOI:** 10.1186/s12955-020-01320-4

**Published:** 2020-03-26

**Authors:** Gian Paolo Morgano, Francesca Fulceri, Franco Nardocci, Corrado Barbui, Giovanni Ostuzzi, Davide Papola, Laura Maria Fatta, Alice Josephine Fauci, Daniela Coclite, Antonello Napoletano, Franco De Crescenzo, Gian Loreto D’Alò, Laura Amato, Michela Cinquini, Primiano Iannone, Holger Jens Schünemann, Maria Luisa Scattoni, on behalf of the Italian National Institute of Health guideline working group on Autism Spectrum Disorder, Maurizio Arduino, Cristina Bellosio, Sandra Biasci, Serafino Buono, Corrado Cappa, Concetta Cordò, Emanuela Di Tommaso, Clelia Maria Duff, Claudia Felici, Angelo Massagli, Massimo Molteni, Laura Reali, Raffaella Tancredi, Giovanni Valeri, Lorella Venturini, Alessandro Zuddas, Mauro Andreoli, Consuelo Bergamin, Marco Bertelli, Danilo Catania, Roberto Cavagnola, Pietro Cirrincione, Serafino Corti, Marusca Crognale, Raffaella Faggioli, Anna Maria Giogoli, Serenella Grittani, Roberto Keller, Pasqualina Pace, Pierluigi Politi, Fabrizio Starace, Marco Valenti, Sara Balduzzi, Michele Basile, Fabio Cruciani, Roberto D’Amico, Marina Davoli, Marien Gonzalez Lorenzo, Silvia Minozzi, Zuzana Mitrova, Ivan Moschetti, Vanna Pistotti, Rosella Saulle, Simona Vecchi

**Affiliations:** 1 Michael G DeGroote Cochrane Canada Centre and McMaster GRADE Centres, Department of Health Research Methods, Evidence and Impact (formerly Clinical Epidemiology and Biostatistics), McMaster University, 1280 Main Street West, Hamilton, Canada; 2 Research Coordination and Support Service, Istituto Superiore di Sanità, Viale Regina Elena 299, 00161 Rome, Italy; 3 WHO Collaborating Centre for Research and Training in Mental Health and Service Evaluation, Department of Neuroscience, Biomedicine and Movement Sciences, Section of Psychiatry, University of Verona, Verona, Italy; 4 Department of Clinical, Neuro and Developmental Psychology; Faculty of Behavioural and Movement Sciences, Vrije Universiteit, Amsterdam, The Netherlands; 5 Centro Eccellenza Clinica, Qualità e Sicurezza delle Cure, Istituto Superiore di Sanità, Viale Regina Elena 299, 00161 Rome, Italy; 6 Department of Epidemiology, Lazio Regional Health Service, Via Cristoforo Colombo, 112, 00154 Rome, Italy; 7 Department of Psychiatry, University of Oxford, Oxford, UK; 8 School of Hygiene and Preventive Medicine, University of Rome Tor Vergata, Rome, Italy; 9 Mario Negri Institute for Pharmacological Research IRCCS, Via Giuseppe La Masa 19, 20156 Milan, Italy; 10 Department of Medicine, McMaster University, Hamilton, Canada

**Keywords:** Autism Spectrum disorder, Italian National Institute of health, Italian national guidelines system, GRADE approach, Healthcare decision, Diagnosis, Treatment, Recommendations, Guideline

## Abstract

**Background:**

Autism Spectrum Disorder (ASD) is a neuro-developmental disorder that affects communication and behavior with a prevalence of approximately 1% worldwide. Health outcomes of interventions for ASD are largely Participant Reported Outcomes (PROs). Specific guidelines can help support the best care for people with ASD to optimize these health outcomes but they have to adhere to standards for their development to be trustworthy.

**Objective:**

The goal of this article is to describe the new methodological standards of the Italian National Institute of Health and novel aspects of this guideline development process. This article will serve as a reference standard for future guideline development in the Italian setting.

**Methods:**

We applied the new standards of the Italian National Institute of Health to the two guidelines on diagnosis and management of children/adolescents and adults with ASD, with a focus on the scoping, panel composition, management of conflict of interest, generation and prioritization of research questions, early stakeholders’ involvement, and PROs. Recommendations are based on the Grading of Recommendations Assessment, Development and Evaluation (GRADE) Evidence-to-Decision frameworks.

**Results:**

Following a public application process, the ISS established two multidisciplinary panels including people with ASD and/or their caregivers. Seventy-nine research questions were identified as potentially relevant for the guideline on children and adolescents with ASD and 31 for the one on adults with ASD. Questions deemed to have the highest priority were selected for inclusion in the guidelines. Other stakeholders valued their early involvement in the process which will largely focus on PROs. The panels then successfully piloted the development of recommendations using the methodological standards and process set by the ISS with a focus on PROs.

**Conclusions:**

In this article, we describe the development of practice guidelines that focus on PROs for the diagnosis and management of ASD based on novel methods for question prioritization and stakeholder involvement. The recommendations allow for the adoption or adaptation to international settings.

## Introduction

Clinical Practice Guidelines (CPGs) are statements containing recommendations for clinical practice or public health policy. A recommendation describes, for the intended end-user of the guideline, what he or she can or should do in specific situations to achieve the best health outcomes possible, individually or collectively [1]. Besides their primary objective to improve health outcomes through the promotion of evidence-based care and clinical pathways, CPGs also serve as a resource for patients, caregivers, policy-makers, researchers and regulatory bodies.

### Clinical practice guidelines in Italy

Following novel national regulations on responsibilities of healthcare professionals [2], the Italian National Institute of Health (Istituto Superiore di Sanità, ISS) through the recently instituted Centre for Clinical Excellence, Quality and Safety of Care (Centro Nazionale per l’Eccellenza Clinica, la Qualità e la Sicurezza delle Cure, CNEC), is responsible for the governance of the Italian guidelines production process [3]. In this framework, the new Italian National Guidelines System (Sistema Nazionale Linee Guida, SNLG) was established as the pivotal instrument to promote an efficient production mechanism of good quality national guidelines, and the methodological standards recommended for the development and evaluation of CPGs were set. Based on international standards such as the Guidelines International Network (GIN) - McMaster guideline development checklist tool, rigorous methods, combined with systematic and transparent processes, are required by the ISS in its recently published methodological manual for CPGs development [4–6]. These regulations have not been previously applied in Italian national guidelines but are now a requirement in any CPG developed by ISS and, thus, in these two new ISS guidelines for managing ASD.

### Autism spectrum disorder and current guidelines on its diagnosis and treatment in context

The essential behavioral features of ASD are persistent impairment in reciprocal social communication and social interaction and restricted, repetitive patterns of behavior, interests, or activities. These core symptoms are present from early childhood and limit or impair everyday functioning [7], are extremely heterogeneous both in terms of complexity and severity and vary over time. Recent systematic review and large observational research reported a prevalence of ASD in adults ranging from 0.7 to 1.1% [8, 9], while a recent study performed by the National Observatory for ASD (coordinated by ISS and the Ministry of Health) revealed that approximately 1.3% of children in the age range 7 to 9 have been diagnosed with ASD in Italy [10]. People with ASD frequently present co-occurring neurological, psychiatric and medical disorders that must be considered for the organization of the appropriate interventions. Outcomes related to interventions, both from tests and management strategies, are typically reported by people with ASD or caregivers. A considerable number of different approaches to diagnose and manage ASD have been proposed over the last 50 years. This reflects the complexity of this condition, which requires a balance between medical, psychological, social, educational and even ethical and existential needs. Many of these approaches have been object of academic and public debate, often with overt disagreement between researchers, clinicians, people with ASD, family and caregivers, and other stakeholders [11, 12].

These factors represent challenges for the development of evidence-based guidelines in this field. In 2011, the ISS published the first Italian Guideline on ASD entitled *‘The treatment of children and adolescents with ASD’*. [13] The published recommendations have been very controversially debated by professionals, institutions and parents’ associations [14]. In 2015, a new law demanded an update of these guidelines and the Italian Ministry of Health appointed the ISS to coordinate the development of national guidelines on management of ASD throughout the lifespan. As opposed to the previous version, these new guidelines will also include diagnostic questions and provide, separately, recommendations for the population of children and adolescents with ASD and for adults with ASD. Furthermore, these guidelines will have to adhere to new methods outlined by CNEC within the framework of the new SNLG and comply with its innovations such as a policy for the disclosure and management of Conflict Of Interest (COI), transparent stakeholder involvement and adoption of the GRADE approach [15, 16]. Yet, these methods have not been tested in real guideline development in this new legal framework.

### Objectives of this article

This article introduces the methods and approach to guideline development at the ISS, laying out its innovative methods using the example of evidence-based guidelines for the diagnosis and management of ASD with a focus on PROs.

## Methods

The guideline development process was guided by the ISS methodological manual [4] and derived from the GIN-McMaster Guideline Development Checklist [6] (https://cebgrade.mcmaster.ca/guidelinechecklistonline.html). It was intended to meet recommendations for trustworthy guidelines by the National Academy of Medicine (U.S.), formerly known as the Institute of Medicine, the World Health Organization and the GIN [5, 17, 18].

### Scope of the guideline

The Italian national law *‘Provisions on prevention, treatment, and rehabilitation of people with autism spectrum disorders, and assistance to families’* (Law 134, approved by the Italian Government on August 2015) intends to ensure the health, the improvement of living conditions and the inclusion in social and working environments of individuals with ASD. The two guidelines described here will be developed in observance of the law 134. Their scope includes the diagnosis and management of ASD and requires describing the perspective, objectives, target population, and target audience.

### Participants in the process

The guideline working group benefits from the contribution of several teams. We describe their roles and responsibilities here.

The ISS Steering Committee (SC) leads and oversees the development of the guideline, it defines the groups involved (chairs, developers, panel, evidence review team) and supports their productive interaction and it is responsible for the development process including budgeting, the definition of a timeline, and the management of COI. The SC, coordinated by a principal investigator and supported by scientific and technical secretariat, selected two chairs for each guideline: one content expert and one methodological expert. The chairs are included in the SC together with a quality assurance team that ensures the compliance of the development process with the ISS methods and regulations.

The guideline group or panel is responsible for prioritization of questions for the guideline, participation in group meetings and teleconferences, providing input on evidence and contextual factors, reviewing evidence summaries, making judgments and formulating recommendations in final panel meetings, reviewing and writing of final guideline report and support for dissemination [19]. Two separate panels have been selected, each focusing on one of the ASD populations of interest: children/adolescents and adults. Considering that the management of ASD, from diagnosis to the delivery of comprehensive care, involves a heterogeneous group of professionals and competencies, the two panels were designed to be multidisciplinary and geographically representative of the entire Italian territory. Through a public process [20], we invited representatives of fields relevant to the guideline’s scope with at least five years of experience and working for the Italian national healthcare system (either in the local health units or in the university/research hospitals) to voluntarily participate in the guidelines. The invitation included representativeness of people with ASD and/or their caregivers. Based on the analysis of their curriculum vitae, cover letter and years of personal or professional experience in the ASD field, sixteen panel members have been selected. All panel members have been invited to sign a declaration of commitment and confidentiality and fill in the COI form. The guideline methodologists or developers, trained in the GRADE approach and the use of the GRADEpro Guideline Development Tool (GRADEpro, https://gradepro.org), work closely with the guideline panel in prioritizing the relevant questions and outcomes, prepare background documents for the guideline panel and stakeholders, coordinate teleconferences and online voting processes, review comments.

The Evidence Review Team (ERT) searches the literature and produces syntheses of the evidence. Following the GRADE approach, the ERT rates the certainty in the evidence, prepares the GRADE evidence tables and Evidence-to-Decision (EtD) frameworks that the panels use in formulating recommendations.

### Management of conflict of interest

The ISS policy on the management of COI follows the GIN principles for disclosure of interests and management of COI in guidelines [18] and it is described in the ISS manual [4]. According to this policy, those involved in the guideline development, including panel members, the ERT, guideline developers and external referees, had to declare all financial, non-financial, personal and institutional interests relevant to the scope of the guidelines completing a standardized form. The SC evaluated each individual interest based on its nature and type, specificity with respect to the scope of guideline, financial value, period and duration. If a declared interest was deemed to represent a conflict, the following measures for the management of COI were applied: full participation, with public disclosure of interest; partial exclusion (e.g. exclusion from the works related to the declared interest and from the relevant decision-making process); total exclusion. We applied the policy throughout the entire process, including during panel members selection, generation and prioritization of research questions, and participation in the formulation of recommendations. We regularly monitored and updated declarations of COI.

### Opening meeting and training of the guideline panel

The working group met for the first time in a two-day meeting held at the ISS headquarter. The following activities took place: the SC outlined the scope of the guideline; guideline developers presented the existing guidelines on ASD; the working group discussed the resources and time available and agreed to produce recommendations on 16 research questions for each of the two ASD identified populations over an 18-month time period. The guideline quality assurance team presented the ISS policy on COI and collected COI disclosure forms from participants. The ERT introduced the GRADE methodology in two presentations. The first presentation served as introduction to the GRADE constructs of certainty in the evidence and strength of recommendations [21, 22]. The second focused on GRADE evidence tables, GRADE EtD frameworks and the importance of people’s values and preferences in decision-making processes [23–26]. We shared links to training material, including the ISS manual and online resources on the GRADE approach to rating the certainty of evidence and the EtD frameworks to meeting participants. The meeting served for the members of the working group to get to know each other and to commence collaboration.

### Selection of guideline questions

We implemented a two-step approach that allowed panels to identify and agree on the questions to be addressed in the guidelines using the module in GRADEpro that allows for the generation and prioritization of questions and health outcomes [27].

### Generation of questions

Guideline developers drafted a list of strategies and interventions addressed in existing CPGs on the diagnosis and management of ASD [28–31]. We discussed the list during the opening meeting and invited panel members to identify items missing or deemed not applicable to the Italian context. Based on the output of the meeting, subgroups including guideline developers and members of the panel with specific expertise (content experts) generated a list of candidate questions framed using the PICO format (population, intervention, comparator, and outcomes) [32]. To streamline the initial list, questions were organized by category (e.g. questions pertaining to the diagnosis, pharmacological, or psychosocial interventions) and, where appropriate, grouped together. The grouping was applied when interventions were assumed to share similar functioning or having similar effects on health outcomes (e.g. medications belonging to the same drug class) and for similar diagnostic instruments. We presented the list of candidate questions to the groups during two-hour recorded web-based conferences.

### Prioritisation of questions

**Fig. 1:**
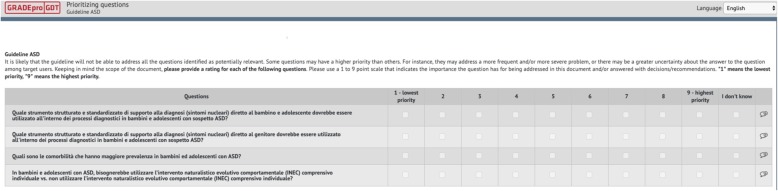
Rating question importance using GRADEpro. GRADEpro interface. Panel members rate the importance of candidate guideline questions on a 1 to 9 scale. Lower ratings are indicative of lower importance

Once the list of candidate questions was finalized, we asked panels to rate the priority of questions on a 1 to 9 scale. We used surveys electronically generated in GRADEpro (Fig. 1) and applied the following criteria: rating of 7 to 9, high priority question - should be addressed in the guideline; rating of 4 to 6, priority question but not of high priority - should be listed as priority in the guideline; rating of 1 to 3, not a priority question - it is acceptable to neither include nor mention it in the guideline.

We invited panel members to consider a brief list of factors that typically influence whether a question is relevant in the context of a CPG (Table 1).

**Table 1:** Factors that should be considered while deciding which are the research questions to be included in a guideline

Factors that influence if a question is important in the context of a guideline	
1. Common question in practice?	
2. Uncertainty in practice?	
3. New evidence to consider?	
4. Variation in practice?	
5. Consequences for resource use/cost?	
6. Not previously or sufficiently addressed?	

We also provided supplementary materials including a glossary of the acronyms used to formulate the questions and articles related to the underpinning theoretical frameworks considered to organize the questions into categories. Following the rating exercise, we presented the results (means, median, minimum and maximum) to the groups in separate two-hour teleconferences using the mean rating score as a ranking criterion. We invited the groups to critically appraise the list and to evaluate its harmony. In particular, we asked to verify if any of the top-rated questions for inclusion could not be considered as exhaustively informative to the reader if not paired with another question that was not rated for inclusion. To achieve harmony, we also organized questions in sensible units, consisting of the smallest recommendation sets that would be informative or required for readers to avoid gaps and achieve rapid dissemination [33]. We used the sensible units to streamline the production and dissemination of recommendations and to create working sub-groups for each, also known as the PICO Responsible Unit (PRU), consisting of content experts and members of the ERT [34].

### Generation of outcomes

**Fig. 2:**
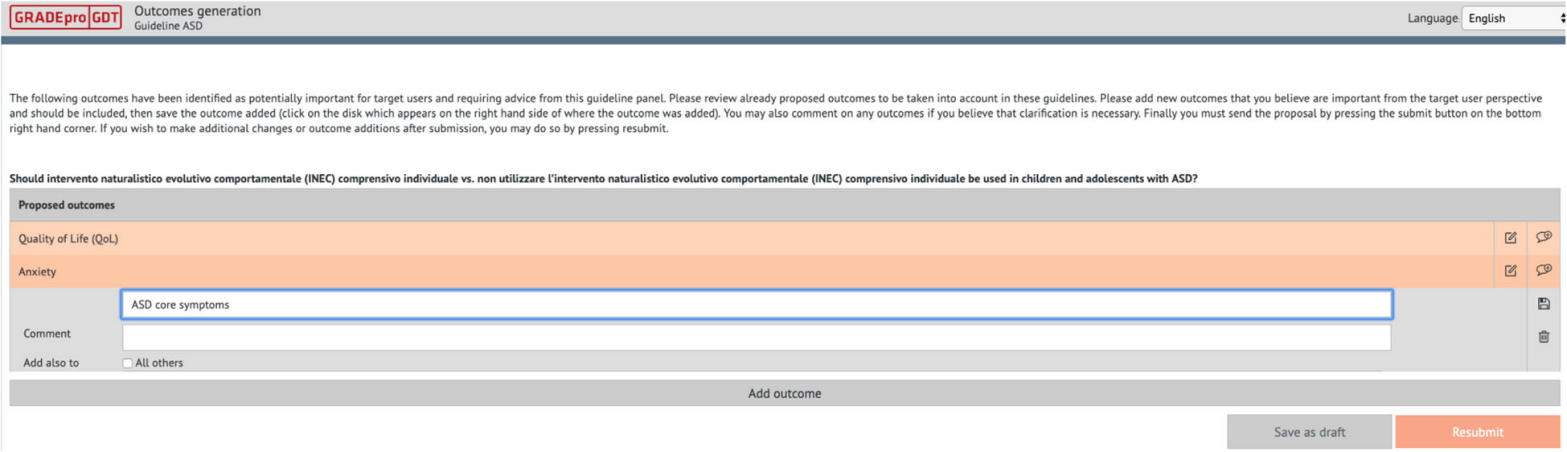
Generation of outcomes using GRADEpro. GRADEpro interface. Panel members suggest, separately for each question, any people-important outcomes that should be considered during the rating of the relative importance of outcomes

To determine the people-important outcomes to be addressed in the syntheses of the evidence, we first engaged the PRUs in drafting descriptions of potentially relevant desirable and undesirable outcomes. We created written definitions of outcomes, known also as *health outcome descriptors*, to reduce the risk of introducing error that could result when panel members have different understanding of the same outcomes. We then sent GRADEpro surveys asking to add, for each question separately, potentially relevant people-important outcomes that were not yet included in the list drafted by the PRUs (Fig. 2).

### Prioritisation of outcomes

**Fig. 3:**
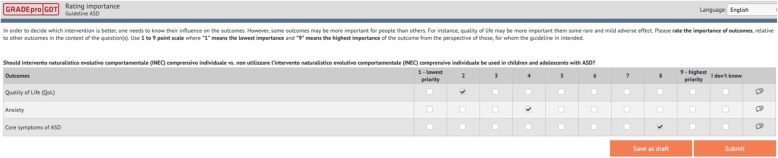
Rating relative importance of outcomes using GRADEpro. GRADEpro interface. Panel members rate the importance of people-important outcomes on a 1 to 9 scale. Lower ratings are indicative of lower importance

We elicited ratings of the relative importance of outcomes on a 1 to 9 scale (Fig. 3) in the corresponding GRADEpro module. We asked the panels to rate outcomes separately for each question using the following criteria: a rating of 7 to 9, the outcome is critical for decision-making; 4 to 6, the outcome is important but not critical for decision-making; 1 to 3, the outcome is of low importance for decision-making [35].

Similar to the question prioritization step, we provided guidance materials on the task and its underpinning concepts, available in additional file 1. We discussed the results of the rating exercise (means, minimum and maximum) in a face-to-face meeting using the mean rating score as the ranking criterion and considered only outcomes rated as critical or important for inclusion in systematic reviews and decision-making during formulation of recommendations. Once the list of outcomes was prioritized, we reached consensus on the final list of questions as described above.

### Stakeholders’ involvement

Public involvement in the development of ISS CPGs is guaranteed through the participation of lay members in the panel as well as also through a public consultation on two key outputs of the process: draft list of guideline questions and draft recommendations. As for the former, we made the list of prioritized questions available for comments by stakeholders who met eligibility criteria. [36] The stakeholders were organized in six categories: scientific societies and health professions associations; family associations and advocacy organizations; national and regional public institutions (e.g. public universities); private institutions (e.g. foundations, private health facilities, private universities); industry (e.g. pharmaceutical companies); public and private research institutes.

Guideline panel members reviewed the comments that were collected electronically using a structured questionnaire (https://piattaformasnlg.iss.it) over a four-week period. Example of questions used in the questionnaire are available in Additional file 2. This early involvement aims at increasing transparency and stakeholder engagement. Similarly, we will invite stakeholders to review and provide comments on the draft recommendations once they will become available. Our dissemination also includes a website (www.osservatorionazionaleautismo.it) where recommendations and the underlying evidence will be available for different user profiles, similar to those of the European Commission Breast Guidelines [34].

### Piloting of the development of recommendations

**Fig. 4:**
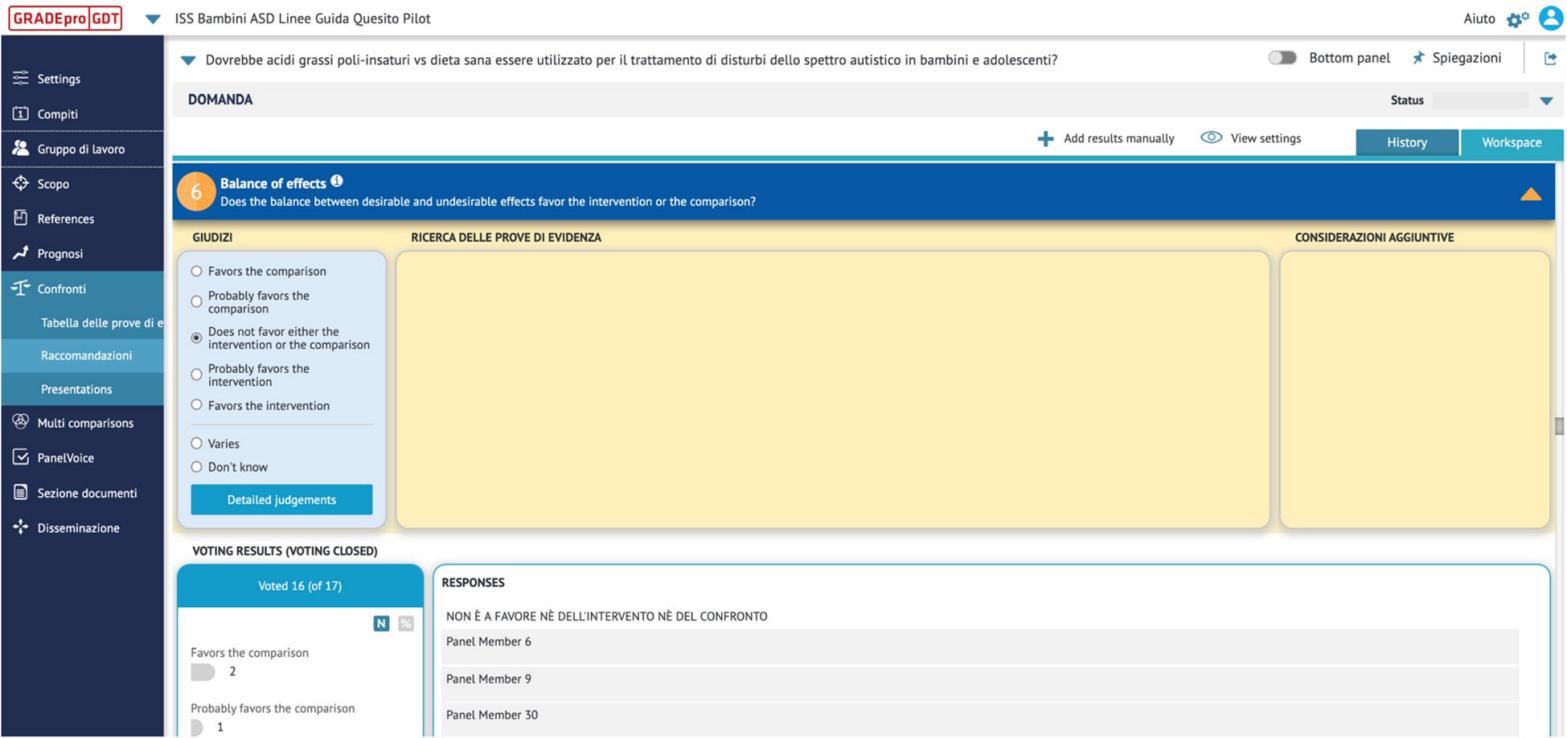
Collection of EtD judgments using PanelVoice. PanelVoice/GRADEpro interface. Judgments on the EtD criteria submitted by panel members are visible to guideline developers and can be used to facilitate the decision-making process online

With the goal to allow the working group to gain experience with the process of making a recommendation and to familiarize with the dynamics typical of guideline panels, we identified two pilot research questions. The ERT conducted systematic reviews and shared the following materials in advance of panel discussion: GRADE Evidence Profiles and a Summary of Findings tables [25, 26] summarizing the effects of the interventions, an EtD framework with structured summaries of the evidence to address each criterion, the list of included and excluded studies, and forest plots where applicable. We piloted the decision-making process using both the in-person and the online approach. In the former, panels met in a meeting room equipped with a u-shaped table, microphones and a recording system. A projector was used by the ERT to present the synthesis of the evidence on a large screen and by the chairs to facilitate discussion and navigate through the various criteria of the EtD. Simultaneously, we also streamed the meeting online using Webex (Cisco Webex, https://www.webex.com/), to allow off-site participation and visualization of content on the screen of panel members’ devices while discussion it. To pilot the online approach, we used the PanelVoice module of GRADEpro (https://gradepro.org/panel-voice/). Through electronic surveys that are integrated in the EtDs, PanelVoice enables guideline developers to facilitate the decision-making process electronically. The process starts with the collection of panel judgments on the EtD criteria (Fig. 4).

**Fig. 5:**
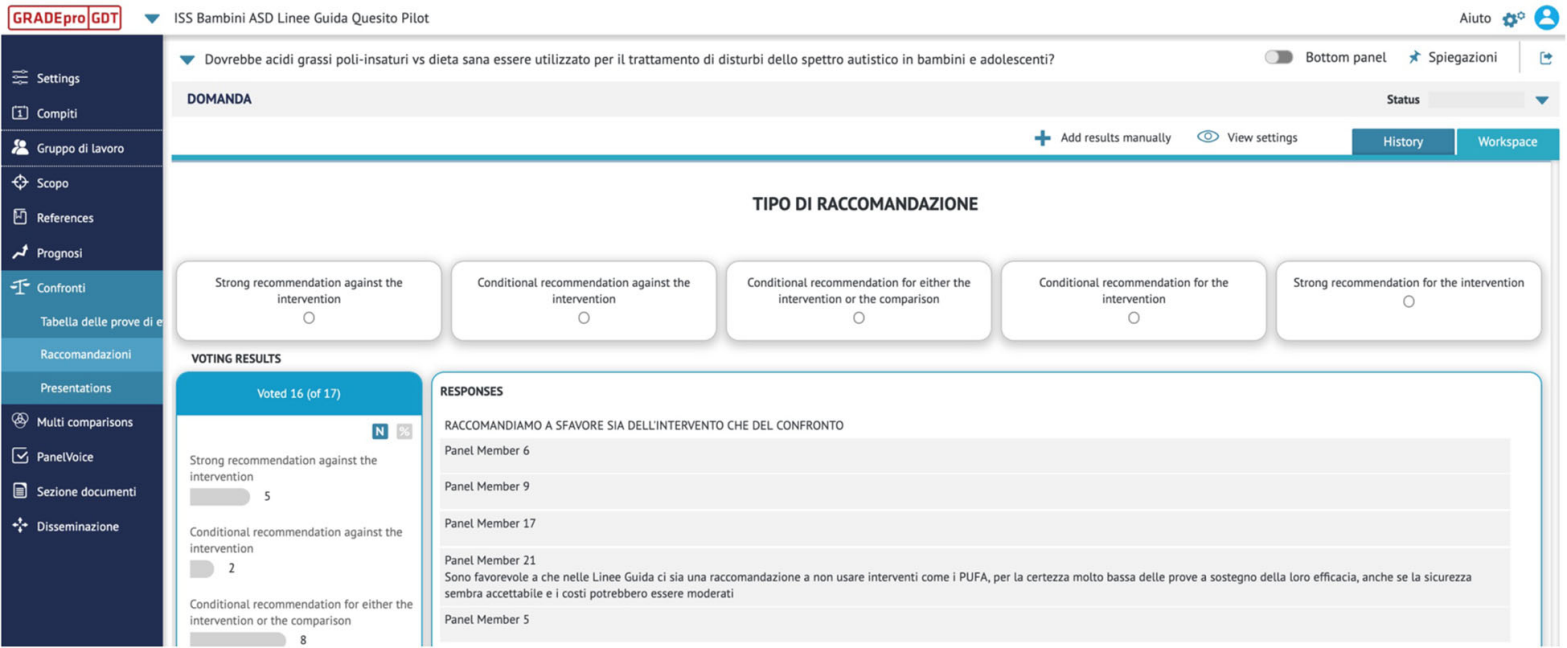
Collection of votes on the strength and direction of recommendations using the GRADEpro/PanelVoice interface. Voting results for the direction and strength of the recommendation are visible to guideline developers and can be used to reach online agreement about the final recommendation

Results of the PanelVoice are reported and agreement reached through email interaction or other necessary. The panel is then asked to decide and agree on the direction and strength of the recommendation and to formulate the statements to be reported in the EtD conclusions section (e.g. justification, implementation considerations, research priorities etc.) (Fig. 5).

## Results

### Composition of the guideline panels

Between June and July 2018, the steering committee received 158 applications for the two multidisciplinary and multi-professional panels of independent experts. Twenty-six applicants were not eligible because employed in private healthcare facilities or universities, had undocumented declared professional competences, their professional profile was not requested in the public selection announcement, or they applied after the submission deadline. Among the 138 who met the requirements, the SC selected 16 applicants per panel on the basis of their professional and personal experience, expertise, healthcare setting (primary, secondary and tertiary care), and geographical representation. Table 2 shows the compositions of the two panels.

**Table 2:** Composition of the guideline working groups

	Children and adolescents	Adults
Expertise	n.	n.
Child Neurologist and Psychiatrist	4	1
Psychiatrist	1	4
Psychologist	2	2
Pshycopharmacologist	1	1
Childhood neuro and psychomotricity therapist	1	–
Speech therapist	1	–
Pedagogues	1	1
Social worker	–	1
Educational therapist	1	1
Occupational therapist	–	2
Expert in the management of healthcare systems	1	1
General practitioner	1	1
Pediatrician	1	–
Methodologist	1	1
Parent of child or adolescents with ASD	2	1
Person with ASD	–	1

### Management of Conflict of interest

The SC reviewed the detailed declarations of interest of all the 158 candidate panelists. None of them was prevented from participating in the panel because of relevant COI, since all the interests declared were considered as manageable through measures such as partial exclusion or public disclosure. Afterwards, the SC evaluated the panelists’ declared and non-declared interests, the latter identified through surveillance of research projects or training activities in which experts are engaged. The SC did not identify any relevant COI that would have prevented guideline panelists from participating in the generation and selection of the research questions addressed.

### Guideline questions

We abstracted interventions and management strategies from previous guidelines into 7 macro-areas to create an initial list: diagnosis and assessment of ASD core-symptoms, diagnosis and assessment of ASD associated features, comorbidities, differential diagnosis, pharmacological interventions, psychosocial interventions, other non-pharmacological interventions. Strategies within the same macro-area were categorized and grouped together by the PRU where applicable. Categorization was based on the target population (e.g. people with ASD versus their caregivers) and on the theory underpinning the interventions. Due to the availability of multiple theoretical frameworks related to non-pharmacological interventions for ASD, the latter categorization presented challenges that we solved through discussion. As for the population of children and adolescents with ASD, the process resulted in a list of 79 questions of which 27 were high-priority, 46 questions important, and 6 questions not important. As for adults, we generated a list of 31 questions of which 21 were high-priority and 10 questions important. For each population, we will develop recommendations to answer 16 research questions whereas all other questions will be mentioned as not prioritized in the guideline. Tables 3 and 4 lists the questions prioritized for inclusion in the guidelines. The lists of all generated questions and their priority ratings are available in Additional file 3.

**Table 3:** List of questions included in the guideline on children and adolescents with ASD

	Question	Macro-area
1	Should structured diagnostic instruments (administered to the children) be added to the clinical assessment from a multidisciplinary team to diagnose ASD core symptoms?	Diagnosis
2	Should structured diagnostic instruments (administered to the parents or caregivers) be added to the clinical assessment from a multidisciplinary team to diagnose ASD core symptoms?	Diagnosis
3	Which are the most prevalent comorbidities in children and adolescents with ASD?	Diagnosis
4	Should INEC comprehensive individual vs no intervention or treatment as usual be used for children and adolescents with ASD?	Psychosocials interventions
5	Should ABA comprehensive vs no intervention or treatment as usual be used for children and adolescents with ASD?	Psychosocials interventions
6	Should Educational comprehensive individual vs no intervention or treatment as usual be used for children and adolescents with ASD?	Psychosocials interventions
7	Should interventions with parents/caregivers vs no intervention or treatment as usual be used for children and adolescents with ASD?	Psychosocials interventions
8	Should INEC focalized vs no intervention or treatment as usual be used for children and adolescents with ASD?	Psychosocials interventions
9	Should ABA focalized vs no intervention or treatment as usual be used for children and adolescents with ASD?	Psychosocials interventions
10	Should INEC focalized vs no intervention or treatment as usual be used for children and adolescents with ASD?	Psychosocials interventions
11	Should mood stabilizers vs no intervention be used in children and adolescents with ASD?	Pharmacological interventions
12	Should SSRIs and/or SNRIs vs no SSRIs and/or SNRIs be used in children and adolescents with ASD?	Pharmacological interventions
13	Should D2 blockers vs no D2 blockers be used in children and adolescents with ASD?	Pharmacological interventions
14	Should psychostimulants and/or atomoxetine vs no psychostimulants and/or atomoxetine be used in children and adolescents with ASD?	Pharmacological interventions
15	Should communicative interventions for social communication and interaction vs no intervention or treatment as usual be used in children and adolescents with ASD?	Other interventions
16	Should interventions for specific behaviours vs no intervention or treatment as usual be used in children and adolescents with ASD?	Other interventions

**Table 4:** List of questions included in the guideline on adults with ASD

	Question	Macro-area
1	Should structured diagnostic instruments be added to routine clinical assessment to diagnose ASD in adults?	Diagnosis
2	Should structured diagnostic instruments to assess psichoeducative and adaptive profile be added to the clinical assessment of the adults with ASD?	Diagnosis
3	Should structured diagnostic instruments to assess neuropsychological and cognitive profile be added to the clinical assessment of the adults with ASD?	Diagnosis
4	Should tests or diagnostic examinations be used to identify psychiatric, neurologic and/or selected medical comorbidities in adults with ASD?	Diagnosis
5	Should standardized instruments to rate the quality of life be used in clinical routine for adults with ASD?	Psychosocials interventions
6	Should standardized preference procedures be used to plan the “life project” of adults with ASD?	Psychosocials interventions
7	Should community-based services and housing support be taken into consideration for adults with ASD?	Psychosocials interventions
8	Should psychoeducative programs be implemented in adults with ASD?	Psychosocials interventions
9	Should information/support campaigns for family members, caregivers and other public figures be accomplished in support of adults with ASD?	Psychosocials interventions
10	Should interventions in support of occupational activities be implemented in adults with ASD?	Psychosocials interventions
11	Should psychological interventions be implemented in adults with ASD?	Psychosocials interventions
12	Should antipsychotics vs no antipsychotics be used in adults with ASD?	Pharmacological interventions
13	Should mood stabilizers vs no mood stabilizers be used in adults with ASD?	Pharmacological interventions
14	Should antidepressants vs no antidepressants be used in adults with ASD?	Pharmacological interventions
15	Should stimulants vs no stimulants be used in adults with ASD?	Pharmacological interventions
16	Should “other drugs” vs no “other drugs” be used in adults with ASD?	Pharmacological interventions

### Outcomes

The panel responsible for children and adolescents with ASD rated ASD core-symptoms as critical outcomes for all research questions. Impairments in social interaction and communication, and restricted and repetitive behaviors were considered as distinct core-symptoms of ASD and rated separately. Other critical and important outcomes included quality of life, adaptive functioning skills, and parenting stress. The panel responsible for adults with ASD prioritized outcomes related to quality of life and outcomes such as social inclusion, level of independency from the caregivers, overall functioning and professional competencies. Other important outcomes included core-symptoms, behavioral disturbances, psychotic symptoms and treatments’ side effects. All outcomes were PROs.

### Stakeholders’ consultation on the research questions

**Fig. 6:**
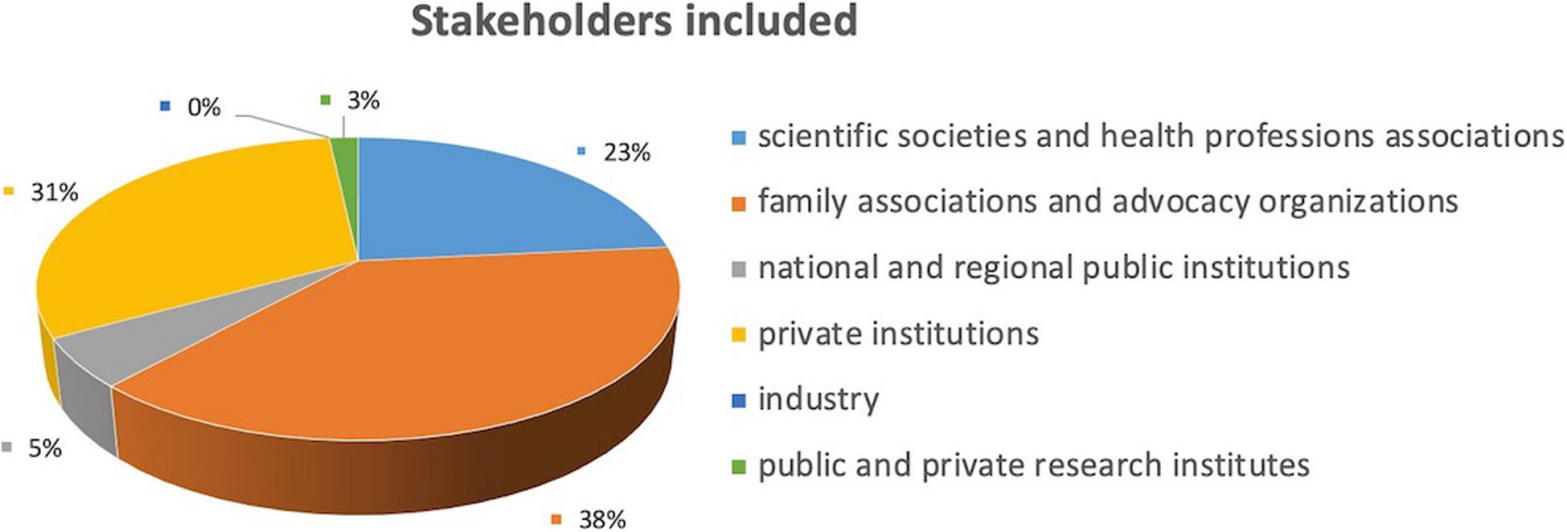
Distribution of registered stakeholders. Pie chart reporting affiliations of the stakeholders participating in the public consultation

Of the 129 stakeholders that requested to comment on the list of questions identified for inclusion in the guideline, 115 met the eligibility criteria. We excluded stakeholders for the following reasons: the application process was not completed or the relationship with healthcare industries was not declared. Figure 6 shows the distribution of registered stakeholders.

The majority of comments pertained to potentially relevant subgroups and outcomes that were not considered in the prioritized guideline questions. Many stakeholders requested clarification regarding the meaning of “standard of care” which was used to phrase some of the questions. Based on the feedback received, we reviewed the comments and improved the wording of research questions and added new sub-groups, where necessary.

## Piloting of the development of recommendations

The questions identified for piloting the process focused on the impact of polyunsaturated fatty acids on PROs in children and adolescents with ASD. The body of evidence consisted of Randomized Controlled Trials (RCTs) and the overall certainty in the estimated effects was rated as very low owing to serious indirectness and very serious imprecision. Based on the very low certainty in the evidence of effects and uncertainty in other judgments on EtD criteria, the panel made conditional recommendations. Further details on the pilot questions, including the EtD framework with panel judgments, are available in Additional file 4.

## Discussion

We have described the methods and processes for guideline development at the ISS using the diagnosis and management of people with ASD as an example. It is the first guideline that follows the new ISS standards and has posed a number of methodological challenges that we addressed using novel guideline development approaches [4]. A challenge particularly relevant to ASD is its focus on PROs in people living in the ASD spectrum and their caregivers.

### Challenges encountered during the development process

The heterogenous composition of the panels, which includes health professionals and stakeholders across a broad spectrum, reflects the complexity of the condition being addressed. The management of such large guideline groups, which encompass different professionals and potentially heterogeneous viewpoints, requires particular ability by chairpersons to conduct effective meetings. Given the broad interest in this guideline by many and diverse stakeholder groups, the process requires maximum possible transparency and we tackled this challenge through the use of GRADE EtDs, the early involvement of key stakeholders and press releases. Applying the ISS COI policy revealed the need for a cultural change. In fact, experts are often not aware that having published on the topic of interest or carried out research or professional activities in the field constitute an interest to be declared. This is important not only for disclosure purposes but also for allowing an assessment of potential conflicts and for determining measures to manage them. We provided guidance to experts in this process to enable them to recognize and declare any circumstance in which a secondary interest could interfere with the impartial performance of their duties, functions and tasks.

### Strengths and innovations of this guideline development process

We created a large multidisciplinary panel which include people with ASD and their caregivers and operate under a transparent policy on COI. Our process for prioritization, using a structured and transparent approach, granted equal voices to panel members and focused on PROs. Our process is supported by independent systematic reviews by the ERT which include an assessment of the certainty in the evidence according to the GRADE approach. We used health outcome descriptors to minimize the bias and improve the overall transparency of the process. Using the GRADE EtD framework, criteria and judgments that yield recommendations are transparent and allow targeting to different user profiles [34]. Through training and piloting exercises, we allowed the panel acquired familiarity with the GRADE approach, the use of the EtD framework, and the summaries of evidence provided to make informed judgments and reach recommendations.

We used information technology to streamline the development process and improve efficiency. Indeed, web-based decision-making and communication tools such as of GRADEpro, StarLeaf, and Webex facilitated work logistics and decreased costs associated with in-person meetings while increasing panel members’ involvement. We promoted stakeholders’ involvement from early stages of the process. The ISS SNLG web platform ensured a transparent and participative process in which stakeholders are empowered to provide valuable feedback in several phases of the process.

### Limitations of this guideline development process

Guideline development requires advanced methodological skills and understanding of evidence. Although panel members are not formally required to know the details of methodology, they must get acquainted with the relevant principles in order to understand the process flow; an ability which demands appropriate training. Human and time resources to develop the syntheses of the evidence that are used to inform the guideline are a very relevant component of the development process but these resources are small compared to the cost of treatment and primary research in this area.

## Conclusions

We have described the new Italian national guideline development process during its first application in recommendations about the diagnosis and management of ASD. The process seems feasible and acceptable to key stakeholders, including guideline panel members, those synthesizing the evidence and the public. The guideline working group is now developing recommendations that will be disseminated and adopted in Italy. This guideline aims to serve as a reference standard for future guideline development in the Italian setting, and it will allow the adoption or adaptation to various settings, including international jurisdictions.

## Supplementary information

**Additional file 1.** Interpretation of ratings for research questions and outcomes.

**Additional file 2.** Example of questions included in the questionnaire for stakeholders.

**Additional file 3.** Ratings of research questions for the ISS ASD guidelines.

**Additional file 4.** EtD for the research question used to pilot the process in-person.

## Data Availability

Data sharing is not applicable to this article as no datasets were generated or analyzed during the current study.

## Abbreviations

ASD: Autism Spectrum Disorder
PROs: Participant Reported Outcomes
GRADE: Grading of Recommendations Assessment, Development and Evaluation
CPGs: Clinical Practice Guidelines
ISS: Istituto Superiore di Sanità (Italian National Institute of Health)
CNEC: Centro Nazionale per l’Eccellenza Clinica, la Qualità e la Sicurezza delle Cure (Centre for Clinical Excellence, Quality and Safety of Care)
SNLG: Sistema Nazionale Linee Guida (Italian National Guidelines System)
COI: Conflict of Interest
GIN: Guidelines International Network
GRADEpro GDT: GRADEpro Guideline Development Tool
ERT: Evidence Review Team
EtD: Evidence-to-Decision
PICO: population, intervention, comparator, outcomes
PRU: PICO Responsible Unit
RCT: Randomized Controlled Trial
