# Supplementary material for: Introduction and methods of the evidence-based guidelines for the diagnosis and management of autism spectrum disorder by the Italian National Institute of Health
Source: Health Qual Life Outcomes. 2020 Mar 26;18:81. doi: 10.1186/s12955-020-01320-4 (PMC7098105; doi:10.1186/s12955-020-01320-4)
Supplement: Supplementary file 1 — Additional file 1. Interpretation of ratings for research questions and outcomes. [file 12955_2020_1320_MOESM1_ESM.docx]

**Additional file 1 .** Interpretations of ratings for research questions and outcomes

| Interpretation of ratings on the 1 to 9 scale for prioritisation of questions | Interpretation of ratings on the 1 to 9 scale for prioritisation of outcomes |
| --- | --- |
| - **7 to 9** the question has high priority. It should be addressed in the guideline. - **4 to 6** the question is considered a priority but not having high priority relative to other questions. It should be listed as a priority question but not addressed in the guideline. - **1 to 3** the question is not a priority. It is acceptable to neither include nor mention it in the guideline. | - **7 to 9** the outcome is *critical* for decision making - **4 to 6** the outcome is *important* but not critical for decision making - **1 to 3** the outcome is of *low importance* |
