# Supplementary material for: Introduction and methods of the evidence-based guidelines for the diagnosis and management of autism spectrum disorder by the Italian National Institute of Health
Source: Health Qual Life Outcomes. 2020 Mar 26;18:81. doi: 10.1186/s12955-020-01320-4 (PMC7098105; doi:10.1186/s12955-020-01320-4)
Supplement: Supplementary file 2 — Additional file 2. Example of questions included in the questionnaire for stakeholders. [file 12955_2020_1320_MOESM2_ESM.docx]

**Additional file 2.** Example of questions included in the questionnaire for stakeholders

| **Example of questions included in the questionnaire for stakeholders** |
| --- |
| Are the population and its sub-populations clearly described? Is there any relevant sub-group of the population that was not listed? |
| Is the intervention clearly described? Is there any relevant intervention related to the type of interventions being addressed that was not listed? |
| Is the comparison clearly described? Is there any relevant comparison related to the type of interventions being addressed that was not listed? |
| Are the outcomes clearly described? Is there any relevant outcome that was not listed? |
