# Supplementary material for: Introduction and methods of the evidence-based guidelines for the diagnosis and management of autism spectrum disorder by the Italian National Institute of Health
Source: Health Qual Life Outcomes. 2020 Mar 26;18:81. doi: 10.1186/s12955-020-01320-4 (PMC7098105; doi:10.1186/s12955-020-01320-4)
Supplement: Supplementary file 3 — Additional file 3. Ratings of research questions for the ISS ASD guidelines. [file 12955_2020_1320_MOESM3_ESM.docx]

**Additional file 3**. Ratings of research questions for the ISS ASD guidelines

Questions on children and adolescents with ASD

| Rank | **Section** | **Question** | **Mean** |
| --- | --- | --- | --- |
| 1 | TREATMENT | [Sezione 5 - Psicosociali/Genitori-Caregivers] Per bambini e adolescenti con ASD, bisognerebbe utilizzare parent training vs. intervento di sostegno psicoeducativo con i genitori/caregiver? | **8.43** |
| 2 | TREATMENT | [Sezione 5 - Psicosociali/Comprensivi/Bambino] In bambini e adolescenti con ASD, bisognerebbe utilizzare l’intervento INEC comprensivo individuale vs. nessun intervento o treatment as usual? | **8.31** |
| 3 | TREATMENT | [Sezione 5 - Altri Interventi] In bambini e adolescenti con ASD, bisognerebbe utilizzare interventi comunicativi per la comunicazione sociale e l’interazione (include social stories, interventi che utilizzano le nuove tecnologie, interventi mediati dai coetanei, training sulla teoria della mente) nessun intervento o treatment as usual? Se si quali? | **8.21** |
| 4 | DIAGNOSIS | [Sezione 1- Sintomi Core/Bambino] Per la diagnosi di ASD (sintomi core) in bambini e adolescenti è utile l'utilizzo di strumenti strutturati standardizzati di supporto alla diagnosi diretti al bambino, in aggiunta all’osservazione e al colloquio clinico (OCC), verso il solo OCC? Se si, quale? | **8.20** |
| 5 | TREATMENT | [Sezione 5 - Psicosociali/Comprensivi/Bambino] In bambini e adolescenti con ASD, bisognerebbe utilizzare l’intervento Evolutivo comprensivo individuale vs. nessun intervento o treatment as usual? | **8.08** |
| 6 | TREATMENT | [Sezione 5 - Psicosociali/Comprensivi/Bambino] In bambini e adolescenti con ASD, bisognerebbe utilizzare l’intervento ABA comprensivo individuale vs. nessun intervento o treatment as usual? | **8.00** |
| 7 | DIAGNOSIS | [Sezione 1- Sintomi Core/Genitori] Per la diagnosi di disturbo dello spettro autistico (ASD) (sintomi core) in bambini e adolescenti è utile l'utilizzo di strumenti strutturati standardizzati di supporto alla diagnosi diretti ai genitori, in aggiunta all’osservazione e al colloquio clinico (OCC), verso il solo OCC? Se si, quale? | **7.93** |
| 8 | TREATMENT | [Sezione 5 - Psicosociali/Genitori-Caregivers] Per bambini e adolescenti con ASD, bisognerebbe utilizzare trattamenti con i genitori/caregiver vs. nessun intervento o treatment as usual? Se si, quale? | **7.93** |
| 9 | TREATMENT | In bambini e adolescenti con ASD, bisognerebbe utilizzare gli stabilizzanti dell’umore vs. placebo o nessun intervento? | **7.92** |
| 10 | TREATMENT | [Sezione 5 - Altri Interventi] In bambini e adolescenti con ASD, bisognerebbe utilizzare interventi per comportamenti specifici (include programmi sulle abilità sociali, social skill group (nice), lego therapy, sulp, junior detective training program) nessun intervento o treatment as usual? Se si quali? | **7.85** |
| 11 | TREATMENT | [Sezione 5 - Psicosociali/Focalizzati/Bambino] In bambini e adolescenti con ASD, bisognerebbe utilizzare l’intervento INEC focalizzato individuale vs. nessun intervento o treatment as usual? | **7.83** |
| 12 | DIAGNOSIS | [Sezione 3 - Comorbilità] Quali sono le comorbilità che hanno maggiore prevalenza in bambini ed adolescenti con ASD e che dovrebbero essere prese in considerazione durante il processo di valutazione? | **7.60** |
| 13 | TREATMENT | In bambini e adolescenti con ASD, bisognerebbe utilizzare gli inibitori del reuptake della serotonina (SSRI) vs. placebo o nessun intervento? | **7.54** |
| 14 | TREATMENT | In bambini e adolescenti con ASD, bisognerebbe utilizzare gli antipsicotici vs. placebo o nessun intervento? | **7.54** |
| 15 | TREATMENT | [Sezione 5 - Psicosociali/Focalizzati/Bambino] In bambini e adolescenti con ASD, bisognerebbe utilizzare l’intervento ABA focalizzato individuale vs. nessun intervento o treatment as usual? | **7.50** |
| 16 | TREATMENT | [Sezione 5 - Psicosociali/Comprensivi/Bambino] In bambini e adolescenti con ASD, bisognerebbe utilizzare l’intervento Educativo comprensivo individuale vs. nessun intervento o treatment as usual? | **7.46** |
| 17 | TREATMENT | In bambini e adolescenti con ASD, bisognerebbe utilizzare gli inibitori del reuptake della norepinefrina vs. placebo o nessun intervento? | **7.38** |
| 18 | TREATMENT | [Sezione 5 - Psicosociali/Focalizzati/Bambino] In bambini e adolescenti con ASD, bisognerebbe utilizzare l’intervento Evolutivo focalizzato individuale vs. nessun intervento o treatment as usual? | **7.36** |
| 19 | TREATMENT | [Sezione 5 - Altri Interventi] In bambini e adolescenti con ASD, bisognerebbe utilizzare la Cognitive Behavioural Therapy (CBT) vs. nessun intervento o treatment as usual? | **7.36** |
| 20 | DIAGNOSIS | [Sezione 2.1 - Sintomi Non-Core/Cognitivo/Bambino] Per la diagnosi di ASD (sintomi non core_dominio cognitivo) in bambini e adolescenti è utile l'utilizzo di strumenti strutturati standardizzati di supporto alla diagnosi diretti al bambino, in aggiunta all’osservazione e al colloquio clinico (OCC), verso il solo OCC? Se si, quale? | **7.33** |
| 21 | DIAGNOSIS | [Sezione 4 - Diagnosi Differenziale] In bambini e adolescenti che vengono riferiti per sospetto ASD, quali patologie, oltre l'ASD, vengono maggiormente diagnosticate? | **7.27** |
| 22 | DIAGNOSIS | [Sezione 2.2 - Valutazione Globale/Neuropsicologico/Bambino] Per la valutazione globale (dominio neuropsicologico) in bambini e adolescenti con ASD è utile l'utilizzo di strumenti strutturati standardizzati di supporto alla diagnosi diretti al bambino, in aggiunta all’osservazione e al colloquio clinico (OCC), verso il solo OCC? Se si, quale? | **7.21** |
| 23 | DIAGNOSIS | [Sezione 1- Sintomi Core] Per la diagnosi di ASD (sintomi core) in bambini e adolescenti, l'utilizzo combinato di ADOS e ADI-R offre maggiore accuratezza se confrontato all'uso singolo dei due strumenti? | **7.20** |
| 24 | TREATMENT | In bambini e adolescenti con ASD, bisognerebbe utilizzare gli inibitori del reuptake della serotonina e noradrenalina (SNRI) vs. placebo o nessun intervento? | **7.18** |
| 25 | DIAGNOSIS | [Sezione 2.1 - Sintomi Non-Core/Adattivo/Bambino] Per la diagnosi di ASD (sintomi non core_dominio adattivo/diretto al bambino) in bambini e adolescenti è utile l'utilizzo di di Vineland Adaptive Behavior Scales (VABS), in aggiunta all’osservazione e al colloquio clinico (OCC), verso il solo OCC? | **7.14** |
| 26 | TREATMENT | In bambini e adolescenti con ASD, bisognerebbe utilizzare gli psicostimolanti vs. placebo o nessun intervento? | **7.08** |
| 27 | TREATMENT | [Sezione 6 - Terapie del sonno] In bambini e adolescenti con ASD, bisognerebbe utilizzare trattamenti per la gestione del sonno (include CBT, melatonina e invio ad uno specialista del sonno) vs. placebo o nessun intervento? | **7.07** |
| 28 | DIAGNOSIS | [Sezione 2.2 - Valutazione Globale/Comorbilità/Genitori] Per la valutazione globale (dominio comorbilità/diretto ai genitori) in bambini e adolescenti con ASD è utile l'utilizzo della Child Behaviour Checklist (CBCL), in aggiunta all’osservazione e al colloquio clinico (OCC), verso il solo OCC? | **6.87** |
| 29 | DIAGNOSIS | [Sezione 2.1 - Sintomi Non-Core/Adattivo/Genitori] Per la diagnosi di ASD (sintomi non core_dominio adattivo) in bambini e adolescenti è utile l'utilizzo di strumenti strutturati di supporto standardizzati alla diagnosi diretti ai genitori, in aggiunta all’osservazione e al colloquio clinico (OCC), verso il solo OCC? Se si, quale? | **6.86** |
| 30 | TREATMENT | [Sezione 5 - Psicosociali/Comprensivi/Bambino] In bambini e adolescenti con ASD, bisognerebbe utilizzare l’intervento INEC comprensivo individuale vs. Evolutivo comprensivo individuale? | **6.85** |
| 31 | TREATMENT | [Sezione 5 - Psicosociali/Focalizzati/Bambino] In bambini e adolescenti con ASD, bisognerebbe utilizzare l’intervento INEC focalizzato individuale vs. Evolutivo focalizzato individuale? | **6.77** |
| 32 | DIAGNOSIS | [Sezione 2.1 - Sintomi Non-Core/Linguaggio/Genitori] Per la diagnosi di ASD (sintomi non core_dominio linguaggio/diretto ai genitori) in bambini e adolescenti è utile l'utilizzo delle MacArthur-Bates Communicative Development Inventories (MB-CDIs), in aggiunta all’osservazione e al colloquio clinico (OCC), verso il solo OCC? | **6.73** |
| 33 | DIAGNOSIS | [Sezione 2.1 - Sintomi Non-Core/Linguaggio/Bambino] Per la diagnosi di ASD (sintomi non core_dominio linguaggio) in bambini e adolescenti è utile l'utilizzo di strumenti strutturati standardizzati di supporto alla diagnosi diretti al bambino, in aggiunta all’osservazione e al colloquio clinico (OCC), verso il solo OCC? Se si, quale? | **6.64** |
| 34 | DIAGNOSIS | [Sezione 2.1 - Sintomi Non-Core/Adattivo/Educatori] Per la diagnosi di ASD (sintomi non core_dominio adattivo/diretto agli educatori) in bambini e adolescenti è utile l'utilizzo di Adaptive Behavior Assessment System (ABAS), in aggiunta all’osservazione e al colloquio clinico (OCC), verso il solo OCC? | **6.64** |
| 35 | TREATMENT | [Sezione 5 - Psicosociali/Comprensivi/Bambino] In bambini e adolescenti con ASD, bisognerebbe utilizzare l’intervento ABA comprensivo individuale vs. Evolutivo comprensivo individuale? | **6.62** |
| 36 | DIAGNOSIS | [Sezione 2.2 - Valutazione Globale/Contesto di vita/Educatori] Per la valutazione globale (contesto di vita/diretto agli educatori) in bambini e adolescenti con ASD è utile l'utilizzo di Vineland Adaptive Behavior Scales (VABS), in aggiunta all’osservazione e al colloquio clinico (OCC), verso il solo OCC? | **6.60** |
| 37 | TREATMENT | [Sezione 5 - Psicosociali/Comprensivi/Bambino] In bambini e adolescenti con ASD, bisognerebbe utilizzare l’intervento ABA comprensivo individuale vs. INEC comprensivo individuale? | **6.54** |
| 38 | TREATMENT | [Sezione 5 - Psicosociali/Comprensivi/Bambino] In bambini e adolescenti con ASD, bisognerebbe utilizzare l’intervento INEC comprensivo individuale vs. Educativo comprensivo individuale? | **6.54** |
| 39 | TREATMENT | [Sezione 5 - Altri Interventi] In bambini e adolescenti con ASD, bisognerebbe utilizzare interventi di integrazione sensoriale vs. nessun intervento o treatment as usual? | **6.29** |
| 40 | TREATMENT | [Sezione 5 - Psicosociali/Comprensivi/Bambino] In bambini e adolescenti con ASD, bisognerebbe utilizzare l’intervento ABA comprensivo individuale vs. Educativo comprensivo individuale? | **6.23** |
| 41 | TREATMENT | [Sezione 5 - Altri Interventi] In bambini e adolescenti con ASD, bisognerebbe utilizzare la terapia occupazionale vs. nessun intervento o treatment as usual? | **6.21** |
| 42 | TREATMENT | [Sezione 5 - Psicosociali/Focalizzati/Bambino] In bambini e adolescenti con ASD, bisognerebbe utilizzare l’intervento ABA focalizzato individuale vs. Evolutivo focalizzato individuale? | **6.14** |
| 43 | DIAGNOSIS | [Sezione 2.2 - Valutazione Globale/Neuropsicologico/Genitori] Per la valutazione globale (dominio neuropsicologico/diretto ai genitori) in bambini e adolescenti con ASD è utile l'utilizzo di Behavior Rating Inventory of Executive Function (BRIEF), in aggiunta all’osservazione e al colloquio clinico (OCC), verso il solo OCC? | **6.08** |
| 44 | DIAGNOSIS | [Sezione 2.2 - Valutazione Globale/Contesto di vita/Genitori] Per la valutazione globale (contesto di vita) in bambini e adolescenti con ASD è utile l'utilizzo di strumenti strutturati standardizzati di supporto alla diagnosi diretti ai genitori, in aggiunta all’osservazione e al colloquio clinico (OCC), verso il solo OCC? Se si, quale? | **6.07** |
| 45 | DIAGNOSIS | [Sezione 1- Sintomi Core] Per la diagnosi di ASD (sintomi core) in bambini e adolescenti, l'utilizzo di strumenti strutturati standardizzati di supporto alla DIAGNOSIS diretti ai genitori (include strumenti diretti ai genitori diversi da ADI-R) offre maggiore accuratezza dell’uso di ADI-R? | **5.93** |
| 46 | DIAGNOSI | [Sezione 2.2 - Valutazione Globale/Comorbilità/Educatori] Per la valutazione globale (dominio comorbidità/diretto agli educatori) in bambini e adolescenti con ASD è utile l'utilizzo del Teacher Report Form (TRF) in aggiunta all’osservazione e al colloquio clinico (OCC), verso il solo OCC? | **5.93** |
| 47 | TREATMENT | [Sezione 5 - Psicosociali/Comprensivi/Bambino] In bambini e adolescenti con ASD, bisognerebbe utilizzare l’intervento Evolutivo comprensivo individuale vs. Educativo comprensivo individuale? | **5.92** |
| 48 | TREATMENT | [Sezione 5 - Psicosociali/Focalizzati/Bambino] In bambini e adolescenti con ASD, bisognerebbe utilizzare l’intervento ABA focalizzato individuale vs. INEC focalizzato individuale? | **5.92** |
| 49 | DIAGNOSIS | [Sezione 2.2 - Valutazione Globale/Comorbilità/Bambino] Per la valutazione globale (dominio comorbidità/diretto al bambino) in bambini e adolescenti con ASD è utile l'utilizzo dello Youth Self Report (YSR) in aggiunta all’osservazione e al colloquio clinico (OCC), verso il solo OCC? | **5.85** |
| 50 | TREATMENT | In bambini e adolescenti con ASD, bisognerebbe utilizzare gli ormoni peptidici (ossitocina, secretina) vs. placebo o nessun intervento? | **5.85** |
| 51 | DIAGNOSIS | [Sezione 1- Sintomi Core/Educatori] Per la diagnosi di ASD (sintomi core) in bambini e adolescenti è utile l'utilizzo di strumenti strutturati standardizzati di supporto alla diagnosi diretti agli educatori, in aggiunta all’osservazione e al colloquio clinico (OCC), verso il solo OCC? Se si, quale? | **5.73** |
| 52 | TREATMENT | [Sezione 6 - Interventi Nutrizionali] In bambini e adolescenti con ASD, bisognerebbe utilizzare acidi grassi poli-insaturi vs. placebo o nessun intervento? | **5.64** |
| 53 | TREATMENT | [Sezione 5 - Altri Interventi] In bambini e adolescenti con ASD, bisognerebbe utilizzare l' Emotion Recognition Training (ERT) vs. nessun intervento o treatment as usual? | **5.62** |
| 54 | TREATMENT | [Sezione 5 - Altri Interventi] In bambini e adolescenti con ASD, bisognerebbe utilizzare il Face Recognition Training (FRT) vs.nessun intervento o treatment as usual? | **5.62** |
| 55 | TREATMENT | In bambini e adolescenti con ASD, bisognerebbe utilizzare gli α agonisti vs. placebo o nessun intervento? | **5.55** |
| 56 | TREATMENT | In bambini e adolescenti con ASD, bisognerebbe utilizzare gli agenti gabaergici vs. placebo o nessun intervento? | **5.54** |
| 57 | TREATMENT | [Sezione 5 - Altri Interventi] In bambini e adolescenti con ASD, bisognerebbe utilizzare la musicoterapia vs. nessun intervento o treatment as usual? | **5.43** |
| 58 | DIAGNOSIS | [Sezione 2.2 - Valutazione Globale/Neuropsicologico/Educatori] Per la valutazione globale (dominio neuropsicologico/diretto agli educatori) in bambini e adolescenti con ASD è utile l'utilizzo di Behavior Rating Inventory of Executive Function (BRIEF), in aggiunta all’osservazione e al colloquio clinico (OCC), verso il solo OCC? | **5.38** |
| 59 | TREATMENT | [Sezione 6 - Interventi Nutrizionali] In bambini e adolescenti con ASD, bisognerebbe utilizzare dieta senza glutine e/o prodotti caseari vs. placebo o nessun intervento? | **5.33** |
| 60 | TREATMENT | [Sezione 5 - Altri Interventi] In bambini e adolescenti con ASD, bisognerebbe utilizzare attività fisica vs. treatment as usual? | **5.23** |
| 61 | TREATMENT | In bambini e adolescenti con ASD, bisognerebbe utilizzare gli antidepressivi triciclici vs. placebo o nessun intervento? | **5.23** |
| 62 | TREATMENT | [Sezione 6 - Interventi Nutrizionali] In bambini e adolescenti con ASD, bisognerebbe utilizzare supplementazione di vitamine e minerali (fatta eccezione per la vitamina k, il ferro e rame) vs. placebo o nessun intervento ? | **5.15** |
| 63 | TREATMENT | In bambini e adolescenti con ASD, bisognerebbe utilizzare agenti glutammatergici vs. placebo o nessun intervento? | **5.08** |
| 64 | TREATMENT | [Sezione 6 - Interventi Nutrizionali] In bambini e adolescenti con ASD, bisognerebbe utilizzare L-Carnosine/L-Carnitine vs. placebo o nessun intervento? | **5.00** |
| 65 | TREATMENT | In bambini e adolescenti con ASD, bisognerebbe utilizzare gli ormoni steroidei vs. placebo o nessun intervento? | **5.00** |
| 66 | TREATMENT | In bambini e adolescenti con ASD, bisognerebbe utilizzare CX516 vs. placebo o nessun intervento? | **5.00** |
| 67 | TREATMENT | In bambini e adolescenti con ASD, bisognerebbe utilizzare gli antistaminici vs. placebo o nessun intervento? | **4.83** |
| 68 | TREATMENT | [Sezione 5 - Altri Interventi] In bambini e adolescenti con ASD, bisognerebbe utilizzare la terapia assistita con gli animali vs. nessun intervento o treatment as usual? Se si quale? | **4.64** |
| 69 | TREATMENT | In bambini e adolescenti con ASD, bisognerebbe utilizzare gli antiossidanti vs. placebo o nessun intervento? | **4.58** |
| 70 | TREATMENT | In bambini e adolescenti con ASD, bisognerebbe utilizzare la minociclina vs. placebo o nessun intervento? | **4.58** |
| 71 | TREATMENT | [Sezione 5 - Altri Interventi] In bambini e adolescenti con ASD, bisognerebbe utilizzare la terapia psicodinamica vs. nessun intervento o treatment as usual? | **4.50** |
| 72 | TREATMENT | [Sezione 6 - Terapie Complementari] In bambini e adolescenti con ASD, bisognerebbe utilizzare neurofeedback vs. nessun intervento o treatment as usual? | **4.46** |
| 73 | TREATMENT | In bambini e adolescenti con ASD, bisognerebbe utilizzare la fenfluramina vs. placebo o nessun intervento? | **4.31** |
| 74 | TREATMENT | [Sezione 5 - Altri Interventi] In bambini e adolescenti con ASD, bisognerebbe utilizzare la comunicazione facilitata vs. nessun intervento o treatment as usual? | **3.93** |
| 75 | TREATMENT | [Sezione 6 - Terapie Complementari] In bambini e adolescenti con ASD, bisognerebbe utilizzare chelazione lungo termine, chelazione breve termine vs. placebo o nessun intervento? | **3.73** |
| 76 | TREATMENT | [Sezione 6 - Terapie Complementari] In bambini e adolescenti con ASD, bisognerebbe utilizzare agopuntura, elettro-agopuntura, agopressione vs. placebo o nessun intervento? | **3.56** |
| 77 | TREATMENT | [Sezione 6 - Terapie Complementari] In bambini e adolescenti con ASD, bisognerebbe utilizzare auditory integration training vs. nessun intervento o treatment as usual? | **3.54** |
| 78 | TREATMENT | [Sezione 6 - Terapie Complementari] In bambini e adolescenti con ASD, bisognerebbe utilizzare kata exercise training vs. nessun intervento o treatment as usual? | **3.50** |
| 79 | TREATMENT | [Sezione 6 - Terapie Complementari] In bambini e adolescenti con ASD, bisognerebbe utilizzare Qigong massage training vs. nessun intervento o treatment as usual? | **3.08** |

Questions on adults with ASD

| ranking | Section | Domanda | **Mean** |
| --- | --- | --- | --- |
| 1 | TREATMENT | Negli adulti con diagnosi di ASD la pianificazione e la valutazione degli interventi basata su quality of life (e altre person-centred outcome measures) vs la pianificazione e la valutazione degli interventi basata su misure di esito tradizionali. non person-centred (es. sintomi. integrità morfologica e di funzionamento. etc) migliora (OUTCOME)? | **8.18** |
| 2 | TREATMENT | Negli adulti con ASD gli interventi psicoeducativi sono efficaci per (OUTCOME). verso l’assenza di interventi psicoeducativi? Se si. quali? | **8.18** |
| 3 | TREATMENT | Negli adulti con ASD i servizi con équipe multidisciplinari specializzate nell’ASD. verso servizi senza équipe multidisciplinare specializzata nell’ASD. sono efficaci per (OUTCOME)? | **8.06** |
| 4 | DIAGNOSIS | Per la diagnosi di ASD negli adulti è utile l'utilizzo di strumenti strutturati di supporto alla diagnosi. in aggiunta all’osservazione e al colloquio clinico (OCC). verso il solo OCC? Se si. quale? | **7.94** |
| 5 | DIAGNOSIS | In adulti con ASD è utile l'utilizzo di strumenti strutturati di valutazione del profilo adattivo in aggiunta all’OCC. verso il solo OCC? Se si. quale? | **7.71** |
| 6 | TREATMENT | Negli adulti con ASD gli interventi di informazione/supporto per familiari sono efficaci per (OUTCOME degli adulti con ASD). rispetto all’assenza di tali interventi? | **7.71** |
| 7 | DIAGNOSIS | In adulti con ASD quali test o esami diagnostici dovrebbero essere effettuati per identificare l’eventuale presenza di co-occorrenze (sia psichiatriche che mediche)? | **7.65** |
| 8 | TREATMENT | Negli adulti con ASD la pianificazione di progetti individualizzati di vita. basati su una procedura standardizzata di assessment delle preferenze. verso una 9pianificazione non basata su una procedura standardizzata di assessment delle preferenze. è efficace per (OUTCOME)? Se si. quali sono le migliori procedure standardizzate di assessment delle preferenze? | **7.65** |
| 9 | TREATMENT | Negli adulti con ASD gli interventi di supporto alle attività occupazionali sono efficaci per (OUTCOME). verso l’assenza di attività occupazionali? Se si. quali? | **7.65** |
| 10 | TREATMENT | Negli adulti con ASD gli interventi di informazione/supporto per caregiver sono efficaci per (OUTCOME degli adulti con ASD). rispetto all’assenza di tali interventi? | **7.59** |
| 11 | TREATMENT | Negli adulti con ASD gli interventi di supporto alla vita autonoma sono efficaci per (OUTCOME). verso l’assenza di interventi di supporto alla vita autonoma? Se si. quali? | **7.53** |
| 12 | DIAGNOSIS | In adulti con ASD è utile l'utilizzo di test o esami diagnostici. in aggiunta all’OCC. per identificare l’eventuale presenza di co-occorrenze (sia psichiatriche che mediche). verso il solo OCC? | **7.41** |
| 13 | TREATMENT | Negli adulti con ASD i servizi con équipe dedicate alla transizione dall’età evolutiva all’età adulta. verso servizi senza équipe dedicate alla transizione dall’età evolutiva all’età adulta. sono efficaci per (OUTCOME)? | **7.35** |
| 14 | TREATMENT | Negli adulti con ASD le psicoterapie sono efficaci per (OUTCOME). verso l’assenza di psicoterapia? Se si. quali? | **7.35** |
| 15 | DIAGNOSIS | In adulti con sospetto di ASD quali test o esami diagnostici dovrebbero essere effettuati. in aggiunta all’OCC. per una corretta diagnosi differenziale con eventuali condizioni mediche o psichiatriche. verso il solo OCC? | **7.35** |
| 16 | TREATMENT | Negli adulti con ASD gli interventi di informazione/supporto per “altre figure” sono efficaci per (OUTCOME degli adulti con ASD). rispetto all’assenza di tali interventi? | **7.29** |
| 17 | DIAGNOSIS | In adulti con ASD è utile l'utilizzo di strumenti strutturati di valutazione del profilo cognitivo in aggiunta all’OCC. verso il solo OCC? Se si. quale? | **7.24** |
| 18 | DIAGNOSIS | In adulti con ASD è utile l'utilizzo di strumenti strutturati di valutazione del profilo neuropsicologico in aggiunta all’OCC. verso il solo OCC? Se si. quale? | **7.24** |
| 19 | TREATMENT | Negli adulti con ASD l’assunzione di farmaci antipsicotici è efficace per (OUTCOME). verso placebo / no treatment? Se si. quali? | **7.13** |
| 20 | TREATMENT | Negli adulti con ASD intraprendere un percorso abitativo. verso non intraprenderlo. è efficace per (OUTCOME)? | **7** |
| 21 | TREATMENT | Negli adulti con ASD l’assunzione di farmaci antiepilettici è efficace per (OUTCOME). verso placebo / no treatment? Se si. quali? | **7** |
| 22 | TREATMENT | Negli adulti con ASD l’assunzione di farmaci antidepressivi è efficace per (OUTCOME). verso placebo / no treatment? Se si. quali? | **6.94** |
| 23 | TREATMENT | Negli adulti con ASD l’assunzione di farmaci stimolanti è efficace per (OUTCOME). verso placebo / no treatment? Se si. quali? | **6.53** |
| 24 | TREATMENT | Negli adulti con ASD l’assunzione di benzodiazepine è efficace per (OUTCOME). verso placebo / no treatment? Se si. quali? | **6.47** |
| 25 | TREATMENT | Negli adulti con ASD l’assunzione di farmaci “affecting cognition" è efficace per (OUTCOME). verso placebo / no treatment? Se si. quali? | **6.29** |
| 26 | TREATMENT | Negli adulti con ASD intraprendere un percorso residenziale. verso non intraprenderlo. è efficace per (OUTCOME)? | **6.18** |
| 27 | TREATMENT | Negli adulti con ASD intraprendere un percorso semi-residenziale. verso non intraprenderlo. è efficace per (OUTCOME)? | **6.12** |
| 28 | TREATMENT | Negli adulti con ASD l’assunzione di terapie ormonali è efficace per (OUTCOME). verso placebo / no treatment? Se si. quali? | **5.87** |
| 29 | TREATMENT | Negli adulti con ASD gli interventi biomedical sono efficaci per (OUTCOME). verso l’assenza di tali interventi? | **5.5** |
| 30 | TREATMENT | Negli adulti con ASD gli interventi sul sonno sono efficaci per (OUTCOME). verso l’assenza di tali interventi? | **5.47** |
| 31 | TREATMENT | Negli adulti con ASD gli interventi nutrizionali sono efficaci per (OUTCOME). verso l’assenza di tali interventi? | **4.94** |
