# Supplementary material for: Introduction and methods of the evidence-based guidelines for the diagnosis and management of autism spectrum disorder by the Italian National Institute of Health
Source: Health Qual Life Outcomes. 2020 Mar 26;18:81. doi: 10.1186/s12955-020-01320-4 (PMC7098105; doi:10.1186/s12955-020-01320-4)
Supplement: Supplementary file 4 — Additional file 4. EtD for the research question used to pilot the process in-person. [file 12955_2020_1320_MOESM4_ESM.docx]

| **Additional file 4.** EtD for the research question used to pilot in-person the process | |
| --- | --- |
| **Si dovrebbero usare gli acidi grassi poli-insaturi vs. non usare gli acidi grassi poli-insaturi for il trattamento di disturbi dello spettro autistico in bambini e adolescenti??** | |
| **PoPOLAZIONE:** | bambini e adolescenti con disturbi dello spettro autistico |
| **Intervento:** | acidi grassi poli-insaturi |
| **Confronto:** | placebo |
| **Esiti principali:** | Discontinuation due to any cause; Iperattività; Qualità del sonno; Autolesionismo; Aggressività; Irritabilità; Ansia; Attenzione; Funzionamento adattivo; Interazione sociale; Interessi e comportamenti ristretti e ripetitivi; Comunicazione; Iperattività e comportamenti dirompenti coesistenti con i sintomi coreith core symptoms; Numero di eventi avversi; |
| **Setting:** | pazienti ambulatoriali |
| **Prospettiva:** | Sistema Sanitario Nazionale Italiano |
| **Background:** |  |
| **Conflitti di interesse** | Noessuno |

# Valutazione

| Problema Il problema è una priorità? | | |
| --- | --- | --- |
| Giudizi | Ricerca delle prove di evidenza | Considerazioni aggiuntive |
| ○ No ○ Probabilmente no ○ Probabilmente si ● Si ○ Varia ○ Non so | Il disturbo dello spettro autistico è caratterizzato da un neurosviluppo anomalo, con alterazioni persistenti dell’interazione sociale, della comunicazione e con interessi e comportamenti ristretti e ripetitivi che causano un funzionamento ridotto, indipendentemente dalla abilità intellettiva (4).  La prevalenza del disturbo dello spettro autistico tra i bambini in Italia è circa 1.35% (dati ISS, unpublished), mentre nel resto dell’Europa varia da 0.63% in Danimarca e Svezia, a 1.16% nel Regno Unito. Negli Stati Uniti la prevalenza è 1.69% attualmente ed è cresciuta molto negli ultimi 20 anni, passando da 0.67% nel 2000 a 1.14% nel 2008 a 1.69% nel 2014 (www.cdc.gov/ncbddd/autism/documents/ASDPrevalenceDataTable2016.pdf). La prevalenza media nel mondo, da studi europei, asiatici ed americani, si attesta tra 1% e 2% (www.cdc.gov/ncbddd/autism/documents/ASDPrevalenceDataTable2016.pdf). Un recente studio italiano, effettuato su 7927 bambini ed adolescenti con diagnosi dello spettro autistico, ha trovato che il rapporto maschi: femmine è di circa 4:1 (5) e che un bambino su due (47.6%) ha anche una disabilità intellettiva, in accordo con la letteratura internazionale (6).  I costi del disturbo dello spettro autistico sono enormi sia per le famiglie che per la società. Una revisione recente negli Stati Uniti e nel Regno Unito ha considerato un costo totale per tutta la vita di circa 2 milioni di euro per supportare un bambino con disturbo dello spettro autistico e disabilità intellettiva e di circa 1.2 milioni di euro per supportare un bambino con disturbo dello spettro autistico in assenza di disabilità intellettiva (7), con costi omogenei tra le due nazioni e ripartiti soprattutto in educazione ed in perdita di lavoro genitoriale. L’autismo nel Regno Unito è la patologia con maggiori costi socio-sanitari, maggiore rispetto alle demenze e maggiore rispetto a patologie tumorali, patologie cardiovascolari ed ictus messe insieme (7). Gli individui con disturbo dello spettro autistico hanno bisogno di sostegno da parte di servizi sanitari e assistenziali, medici, farmacie e ospedali, per tutta la vita. Tuttavia, troppi individui con disturbo dello spettro autistico aspettano anni prima di ottenere una diagnosi attraverso il servizio sanitario nazionale e di ottenere la cura e il supporto di cui hanno bisogno (8). Inoltre, molti individui con disturbo dello spettro autistico continuano ad avere una salute fisica e mentale significativamente peggiore rispetto al pubblico in generale e possono anche essere maggiormente a rischio di morire presto (9). Il disturbo dello spettro autistico è stato dichiarato come una parte fondamentale del sistema sanitario Italiano, la cui cura può essere modellata intorno ai bisogni delle persone (10).  Gli acidi grassi poli-insaturi (PUFA) sono grassi che contengono almeno due doppi legami carbonio-carbonio nella loro catena carbossilica. I PUFA si dividono, secondo la distanza del primo doppio legame dal gruppo metilico posto al termine della molecola, in omega-3, omega-6 e omega-9 (questi ultimi non sono essenziali nell’uomo in quanto possono essere sintetizzati dai carboidrati o da altri acidi grassi). Gli oli di pesce sono ricchi di omega-3, quelli delle piante di omega-6 e due PUFA, l’acido alfalinoleico (omega-3) e l’acido linoleico (omega-6) sono nutrienti essenziali nell’uomo (11).  Il ruolo di EPA e DHA nei disturbi del sistema nervoso centrale è stato ampiamente indagato nelle ultime due decadi (2). EPA e DHA sono fattori importanti nello sviluppo dei sistemi nervoso e immune fetali. EPA e DHA sono importanti componenti dei fosfolipidi e degli esteri di colesterolo delle membrane neuronali, specialmente dei dendriti e delle sinapsi; pertanto il razionale nell’uso di questi agenti nei disordini di natura psichiatrica sarebbe proprio la loro azione primaria nel produrre modificazioni della membrana sinaptica, con implicazioni nella trasmissione e trasduzione del segnale (2). Ad esempio, studi di risonanza magnetica hanno suggerito che una ridotta connessione funzionale di aree cerebrali a lunga distanza è correlata alle difficoltà nelle interazioni sociali nel disturbo dello spettro autistico (1). In ambito psichiatrico, EPA e DHA sono stati sperimentati nella terapia di ADHD, autismo, disturbo bipolare, unipolare, disturbi d’ansia, disturbo ossessivo-compulsivo, aggressività, ostilità, impulsività, disturbo di personalità borderline, uso di sostanze e anoressia nervosa (2). L’ipotesi è che i PUFA possano essere efficaci sui sintomi core dell’autismo e che abbiano un buon profilo di sicurezza. Ultimamente c'è stato un aumento sul mercato della disponibilità di molti farmaci e integratori alimentari ed è aumentato anche il numero di PUFA ad uso pediatrico. Un'altra ipotesi è che l'assunzione indiscriminata ed eccessiva di PUFA potrebbe dare dei problemi di sicurezza ed è fondamentale raccomandarne un uso appropriato (3). |  |
| Effetti desiderabili Quanto considerevoli sono gli effetti desiderabili attesi? | | |
| Giudizi | Ricerca delle prove di evidenza | Considerazioni aggiuntive |
| ○ Irrilevanti ●Piccoli ○ Moderati ○ Grandi ○ Variano ○ Non lo so | È stata effettuata una ricerca sistematica della letteratura sulle banche dati CENTRAL, PubMed/Medline, Embase, PsycINFO, Web Of Science, dalla data della creazione delle rispettive banche dati fino al 30 Ottobre 2018, senza limitazioni di lingua. La strategia di ricerca è disponibile su richiesta.  La selezione degli studi, l’estrazione dei dati, la valutazione del rischio di bias e della certezza dell’evidenza secondo il metodo GRADE è stata effettuata da due revisori in modo indipendente. I risultati degli studi che effettuavano confronti diretti sono stati combinati attraverso meta-analisi *pairwise* usando come misure di risultato il risk ratio (RR) per gli esiti dicotomici e la differenza standardizzata tra medie (SMD) per gli esiti continui, utilizzando per entrambe le misure un modello ad effetti casuali.  **Risultati della ricerca per singole Banche dati**  CENTRAL= 69  Embase= 209  MEDLINE= 153  PsycINFO= 90  Web Of Science= 265  Numero di documenti trovati dopo ricerca sistematica= 786  Numero di documenti trovati dopo la rimozione dei duplicati= 558    La strategia di ricerca utilizzata ha permesso il ritrovamento di 786 documenti, dei quali 228 sono stati rimossi, essendo dei duplicati. Dei 558 documenti da valutare, 24 documenti sono stati valutati in full-text. Di questi, 12 sono stati esclusi. Tra gli esclusi, 4 studi includevano bambini nati pretermine tra i 18 ed i 36 mesi con alto rischio di ASD (21, 22); (23) (24), 3 studi non avevano un gruppo di controllo (25); (26); (27), 1 studio non era un RCT (28), 1 studio era un case report (29). Abbiamo trovato uno studio clinico in corso (ACTRN1265000144516) e 2 studi clinici completati ma di cui non abbiamo ancora i risultati (clinicaltrials.gov identifiers: NCT00577447; NCT02059577).  Infine, 12 documenti, corrispondenti ad un totale di 9 studi (351 partecipanti) sono stati inclusi (20); (30); (31); (12); (13); (14, 15); (16, 17); (18) (19, 1).  Gli studi clinici che comprendevano partecipanti in età prescolare erano 6 (66.6%), mentre 3 studi clinici (33.3%) includevano anche individui adolescenti. La maggior parte degli individui inclusi era di sesso maschile (86.6%). In 7 casi la diagnosi è stata effettuata utilizzando i criteri del DSM-IV, in un caso i criteri del DSM-5, in un altro caso la diagnosi era riportata dai genitori. In 5 casi è stato riportato l’utilizzo di scale per supporto alla diagnosi, tra cui ADI-R, ADOS, CARS, SCQ.  Tra i 9 studi inclusi, 8 studi hanno confrontato i PUFA verso placebo, mentre 1 solo studio ha confrontato i PUFA verso un intervento in cui veniva proposto di seguire una dieta sana. Degli studi, 2 sono stati condotti in Europa, 5 in Nord America, 1 in Asia, 1 in Oceania.  Le scale usate per la misurazione degli esiti includevano la Aberrant Behavior Checklist (ABC), la Behavior Assessment System for Children (BASC), la Expressive Vocabulary Test (EVT) , la Mullen Scales of Early Learning, la Peabody Picture Vocabulary Test (PPVT), la Social Responsiveness Scale (SRS), la Vineland Adaptive Behavior Scale (VABS).  Per quanto riguarda la composizione dei PUFA, in 5 studi clinici era presente una combinazione di acido eicosapentaenoico (EPA) ed acido docosaesaenoico (DHA), mentre in 4 studi era presente solo DHA. Le dosi di EPA variavano tra 693mg e 840mg/die, mentre le dosi di DHA variavano dai 200mg ai 722mg/die.  La modalità di assunzione di PUFA variava grandemente negli studi clinici randomizzati analizzati, con una dose mediana di 1155 mg/die, e dosaggi da un minimo di 200 mg/die (18) ad un massimo di 1540 mg/die (20).  La mediana della durata degli studi clinici era di 12 settimane (range: 6-52).    Per quanto riguarda l'assunzione raccomandata di acidi grassi omega-3 per neonati, l'OMS suggerisce 400 mg per 10 kg di peso corporeo (WHO/FAO Expert Consultation on Diet, undefined) , (Lee, 2013)), mentre l' International Scientific Society of Fatty Acids and Lipids (ISSFAL) suggerisce 350-750 mg ogni 10 kg di peso corporeo (http://www.issfal.org/news-links/resources/publications/PUFAIntakeReccomdFinalReport.pdf, undefined).  Riguardo invece la massima dose tollerabile di omega-3, la Food and Drug Administration (FDA) raccomanda di non assumere più di 3 g/die di EPA e DHA, dei quali fino a 2d/die attraverso i supplementi (National Institutes of Health, undefined) La limitazione giornaliera è importante al fine di limitare l'assunzione di vitamine liposolubili, quali Vitamina A e Vitamina D (Bays, 2007) (Lee, 2013). L’Institute Of Medicine (IOM) non ha stabilito un tolerable Upper Intake Level (UL) per l’assunzione di omega-3, ma ha evidenziato che dosi elevate (più di 900mg/die di EPA più 600 mg/die di DHA) potrebbero ridurre la risposta immunitaria, mentre dosi tra i 2 e i 15 grammi di EPA e/o DHA potrebbero avere effetti negativi sulla coagulazione, favorendo i sanguinamenti (Institute of Medicine et al., undefined). Secondo la European Food Safety Authority (EFSA), invece, la supplementazione con dosi fino a 5g/die di EPA e/o DHA sarebbe sicura, non essendo stati riscontrati effetti collaterali riguardo il sanguinamento e risposta immune (EFSA Panel on Dietetic Products NaA. Scientific opinion on the tolerable upper intake level of eicosapentaenoic acid (EPA) et al., undefined).  Una revisione sistematica recente sottolinea la differenza in materia di sicurezza tra Omega-3 prodotti come nutraceutici rispetto ai farmacologici, sottolineando come i prodotti farmacologici prescritti sono supportati da robusti programmi di sviluppo clinico e di monitoraggio della sicurezza, mentre i prodotti nutraceutici non sono tenuti a dimostrare sicurezza o efficacia prima del marketing (Hilleman D, 2016). I nutraceutici possono anche contenere componenti potenzialmente dannosi, tra cui altri lipidi, colesterolo e tossine e non sono prodotti in Good Manifacturing Practice (GMP). I prodotti farmacologici omega-3 possono contenere DHA ed EPA o EPA ad elevata purezza (Hilleman D, 2016) (Santini A, 2018). Nonostante nei prodotti ittici sia presete metil-mercurio in varie quantità, questo non si dovrebbe ritrovare abitualmente nei supplementi a base di omega-3, in quanto rimosso nel processo di produzione (ConsumerLab.com. Product review: fish oil and omega-3 fatty acid supplements review (including krill, undefined); (National Institutes of Health, undefined)  Oltre alla presa in esame degli studi inclusi, dalla precedente revisione sistematica Cochrane (James S, 2011) abbiamo anche ripreso gli studi osservazionali esclusi, per valutare la presenza di eventuali evidenze aggiuntive sull’accettabilità e la sicurezza della supplementazione con omega-3 negli individui autistici in età pediatrica. Tra gli studi osservazionali, alcuni andavano a valutare la sicurezza dell'assunzione dei PUFA per individui con disturbo dello spettro autistico. In uno studio la supplementazione con PUFA è stata associata ad un aumento dell' iperattività e problemi comportamentali, riferiti dai genitori (Bell JG, 2004).         \| **Esiti** \| **Effetto assoluto anticipato^*^ (95% CI)** \| \| **Effetto relativo (95% CI)** \| **№ dei partecipanti (studi)** \| **Certainty of the evidence (GRADE)** \| **Commenti** \| \| --- \| --- \| --- \| --- \| --- \| --- \| --- \| \| **Rischio con placebo** \| **Rischio con acidi grassi poli-insaturi** \| \| Iperattività \| La media iperattività eran-a \| SMD 0.27 inferiore (0.6 inferiore a 0.06 maggiore) \| - \| 146 (5 RCT) \| ⨁⨁◯◯ BASSA^a^ \|  \| \| Aggressività \| La media aggressività eran-a \| SMD 0.29 inferiore (1.08 inferiore a 0.49 maggiore) \| - \| 25 (1 RCT) \| ⨁⨁◯◯ BASSA^a^ \|  \| \| Irritabilità \| La media irritabilità eran-a \| SMD 0.02 inferiore (0.42 inferiore a 0.38 maggiore) \| - \| 146 (5 RCT) \| ⨁⨁◯◯ BASSA^a^ \|  \| \| Ansia \| La media ansia eran-a \| SMD 1.01 inferiore (1.86 inferiore a 0.17 inferiore) \| - \| 25 (1 RCT) \| ⨁◯◯◯ MOLTO BASSA^a,b^ \|  \| \| Funzionamento adattivo \| La media funzionamento adattivo eran-a \| SMD 0.49 inferiore (1.2 inferiore a 0.22 maggiore) \| - \| 59 (2 RCT) \| ⨁◯◯◯ MOLTO BASSA^a,c,d^ \|  \| \| Interazione sociale \| La media interazione sociale eran-a \| SMD 0.27 maggiore (0.03 inferiore a 0.57 maggiore) \| - \| 172 (4 RCT) \| ⨁◯◯◯ MOLTO BASSA^a,e^ \|  \| \| Interessi e comportamenti ristretti e ripetitivi \| La media interessi e comportamenti ristretti e ripetitivi eran-a \| SMD 0.01 maggiore (0.36 inferiore a 0.39 maggiore) \| - \| 223 (6 RCT) \| ⨁⨁◯◯ BASSA^a^ \|  \| \| Comunicazione \| La media comunicazione era n-a SD \| SMD 0.05 SD inferiore (0.5 inferiore a 0.4 maggiore) \| - \| 223 (6 RCT) \| ⨁⨁◯◯ BASSA^a^ \|  \| \| Numero di eventi avversi \| Popolazione in studio \| \| **RR 1.54** (0.79 a 2.97) \| 157 (5 RCT) \| ⨁⨁◯◯ BASSA^f^ \|  \| \| 132 per 1,000 \| 203 per 1,000 (104 a 391) \|  1. Downgraded of two levels because population size <400 and there is a wide 95%CI, which includes no effect 2. Downgraded of one level because the measure used was the internalizing subscale of the BASC, which only indirectly measures anxiety 3. Downgraded of one level because one study is at high risk for incomplete outcome data and unclear risk for blinding and selective reporting 4. Downgraded of one level, because in one study the "social skills, parents assessed" of the subscale "adaptive skills" of the BASC was extracted 5. Downgraded of one level because in two studies Social interaction was analysed by the "inappropriate speech" subscale of the ABC, which relates more to behaviour and indirectly to social interaction 6. Downgraded of two levels because optimal information size (OIS) not met and there is a wide 95%CI, which includes no effect | Queste regole empiriche si basano sulle analisi di Cohen a riguarda della proporzione dell'effetto: - 0.2 è un piccolo effetto (ad esempio SMD 0.45 corrisponde ad un piccolo effetto); - 0.5 è un effetto moderato (ad esempio, SMD 0.7 corrisponde ad un effetto moderato); - 0,8 è un grande effetto (ad esempio, SMD 0,95 corrisponde ad un grande effetto).    Trivial: Insignificante - 2  Small: Basso - 13  Moderate: Moderato- 2  Large: Grande- 0  Astenuto- 1 |
| Effetti indesiderabili Quanto considerevoli sono gli effetti indesiderabili attesi? | | |
| Giudizi | Ricerca delle prove di evidenza | Considerazioni aggiuntive |
| ○ Grandi ○ Moderati ●Piccoli  ○ Irrilevanti  ○ Variano ○ Non lo so | È stata effettuata una ricerca sistematica della letteratura sulle banche dati CENTRAL, PubMed/Medline, Embase, PsycINFO, Web Of Science, dalla data della creazione delle rispettive banche dati fino al 30 Ottobre 2018, senza limitazioni di lingua. La strategia di ricerca è disponibile su richiesta.  La selezione degli studi, l’estrazione dei dati, la valutazione del rischio di bias e della certezza dell’evidenza secondo il metodo GRADE è stata effettuata da due revisori in modo indipendente. I risultati degli studi che effettuavano confronti diretti sono stati combinati attraverso meta-analisi *pairwise* usando come misure di risultato il risk ratio (RR) per gli esiti dicotomici e la differenza standardizzata tra medie (SMD) per gli esiti continui, utilizzando per entrambe le misure un modello ad effetti casuali.  **Risultati della ricerca per singole Banche dati**  CENTRAL= 69  Embase= 209  MEDLINE= 153  PsycINFO= 90  Web Of Science= 265  Numero di documenti trovati dopo ricerca sistematica= 786  Numero di documenti trovati dopo la rimozione dei duplicati= 558    La strategia di ricerca utilizzata ha permesso il ritrovamento di 786 documenti, dei quali 228 sono stati rimossi, essendo dei duplicati. Dei 558 documenti da valutare, 24 documenti sono stati valutati in full-text. Di questi, 12 sono stati esclusi. Tra gli esclusi, 4 studi includevano bambini nati pretermine tra i 18 ed i 36 mesi con alto rischio di ASD (21, 22); (23) (24), 3 studi non avevano un gruppo di controllo (25); (26); (27), 1 studio non era un RCT (28), 1 studio era un case report (29). Abbiamo trovato uno studio clinico in corso (ACTRN1265000144516) e 2 studi clinici completati ma di cui non abbiamo ancora i risultati (clinicaltrials.gov identifiers: NCT00577447; NCT02059577).  Infine, 12 documenti, corrispondenti ad un totale di 9 studi (351 partecipanti) sono stati inclusi (20); (30); (31); (12); (13); (14, 15); (16, 17); (18) (19, 1).  Gli studi clinici che comprendevano partecipanti in età prescolare erano 6 (66.6%), mentre 3 studi clinici (33.3%) includevano anche individui adolescenti. La maggior parte degli individui inclusi era di sesso maschile (86.6%). In 7 casi la diagnosi è stata effettuata utilizzando i criteri del DSM-IV, in un caso i criteri del DSM-5, in un altro caso la diagnosi era riportata dai genitori. In 5 casi è stato riportato l’utilizzo di scale per supporto alla diagnosi, tra cui ADI-R, ADOS, CARS, SCQ.  Tra i 9 studi inclusi, 8 studi hanno confrontato i PUFA verso placebo, mentre 1 solo studio ha confrontato i PUFA verso un intervento in cui veniva proposto di seguire una dieta sana. Degli studi, 2 sono stati condotti in Europa, 5 in Nord America, 1 in Asia, 1 in Oceania.  Le scale usate per la misurazione degli esiti includevano la Aberrant Behavior Checklist (ABC), la Behavior Assessment System for Children (BASC), la Expressive Vocabulary Test (EVT) , la Mullen Scales of Early Learning, la Peabody Picture Vocabulary Test (PPVT), la Social Responsiveness Scale (SRS), la Vineland Adaptive Behavior Scale (VABS).  Per quanto riguarda la composizione dei PUFA, in 5 studi clinici era presente una combinazione di acido eicosapentaenoico (EPA) ed acido docosaesaenoico (DHA), mentre in 4 studi era presente solo DHA. Le dosi di EPA variavano tra 693mg e 840mg/die, mentre le dosi di DHA variavano dai 200mg ai 722mg/die.  La modalità di assunzione di PUFA variava grandemente negli studi clinici randomizzati analizzati, con una dose mediana di 1155 mg/die, e dosaggi da un minimo di 200 mg/die (18) ad un massimo di 1540 mg/die (20).  La mediana della durata degli studi clinici era di 12 settimane (range: 6-52).    Per quanto riguarda l'assunzione raccomandata di acidi grassi omega-3 per neonati, l'OMS suggerisce 400 mg per 10 kg di peso corporeo (WHO/FAO Expert Consultation on Diet, undefined) , (Lee, 2013)), mentre l' International Scientific Society of Fatty Acids and Lipids (ISSFAL) suggerisce 350-750 mg ogni 10 kg di peso corporeo (http://www.issfal.org/news-links/resources/publications/PUFAIntakeReccomdFinalReport.pdf, undefined).  Riguardo invece la massima dose tollerabile di omega-3, la Food and Drug Administration (FDA) raccomanda di non assumere più di 3 g/die di EPA e DHA, dei quali fino a 2d/die attraverso i supplementi (National Institutes of Health, undefined) La limitazione giornaliera è importante al fine di limitare l'assunzione di vitamine liposolubili, quali Vitamina A e Vitamina D (Bays, 2007) (Lee, 2013). L’Institute Of Medicine (IOM) non ha stabilito un tolerable Upper Intake Level (UL) per l’assunzione di omega-3, ma ha evidenziato che dosi elevate (più di 900mg/die di EPA più 600 mg/die di DHA) potrebbero ridurre la risposta immunitaria, mentre dosi tra i 2 e i 15 grammi di EPA e/o DHA potrebbero avere effetti negativi sulla coagulazione, favorendo i sanguinamenti (Institute of Medicine et al., undefined). Secondo la European Food Safety Authority (EFSA), invece, la supplementazione con dosi fino a 5g/die di EPA e/o DHA sarebbe sicura, non essendo stati riscontrati effetti collaterali riguardo il sanguinamento e risposta immune (EFSA Panel on Dietetic Products NaA. Scientific opinion on the tolerable upper intake level of eicosapentaenoic acid (EPA) et al., undefined).  Una revisione sistematica recente sottolinea la differenza in materia di sicurezza tra Omega-3 prodotti come nutraceutici rispetto ai farmacologici, sottolineando come i prodotti farmacologici prescritti sono supportati da robusti programmi di sviluppo clinico e di monitoraggio della sicurezza, mentre i prodotti nutraceutici non sono tenuti a dimostrare sicurezza o efficacia prima del marketing (Hilleman D, 2016). I nutraceutici possono anche contenere componenti potenzialmente dannosi, tra cui altri lipidi, colesterolo e tossine e non sono prodotti in Good Manifacturing Practice (GMP). I prodotti farmacologici omega-3 possono contenere DHA ed EPA o EPA ad elevata purezza (Hilleman D, 2016) (Santini A, 2018). Nonostante nei prodotti ittici sia presete metil-mercurio in varie quantità, questo non si dovrebbe ritrovare abitualmente nei supplementi a base di omega-3, in quanto rimosso nel processo di produzione (ConsumerLab.com. Product review: fish oil and omega-3 fatty acid supplements review (including krill, undefined); (National Institutes of Health, undefined)  Oltre alla presa in esame degli studi inclusi, dalla precedente revisione sistematica Cochrane (James S, 2011) abbiamo anche ripreso gli studi osservazionali esclusi, per valutare la presenza di eventuali evidenze aggiuntive sull’accettabilità e la sicurezza della supplementazione con omega-3 negli individui autistici in età pediatrica. Tra gli studi osservazionali, alcuni andavano a valutare la sicurezza dell'assunzione dei PUFA per individui con disturbo dello spettro autistico. In uno studio la supplementazione con PUFA è stata associata ad un aumento dell' iperattività e problemi comportamentali, riferiti dai genitori (Bell JG, 2004).         \| **Esiti** \| **Effetto assoluto anticipato^*^ (95% CI)** \| \| **Effetto relativo (95% CI)** \| **№ dei partecipanti (studi)** \| **Certainty of the evidence (GRADE)** \| **Commenti** \| \| --- \| --- \| --- \| --- \| --- \| --- \| --- \| \| **Rischio con placebo** \| **Rischio con acidi grassi poli-insaturi** \| \| Iperattività \| La media iperattività eran-a \| SMD 0.27 inferiore (0.6 inferiore a 0.06 maggiore) \| - \| 146 (5 RCT) \| ⨁⨁◯◯ BASSA^a^ \|  \| \| Aggressività \| La media aggressività eran-a \| SMD 0.29 inferiore (1.08 inferiore a 0.49 maggiore) \| - \| 25 (1 RCT) \| ⨁⨁◯◯ BASSA^a^ \|  \| \| Irritabilità \| La media irritabilità eran-a \| SMD 0.02 inferiore (0.42 inferiore a 0.38 maggiore) \| - \| 146 (5 RCT) \| ⨁⨁◯◯ BASSA^a^ \|  \| \| Ansia \| La media ansia eran-a \| SMD 1.01 inferiore (1.86 inferiore a 0.17 inferiore) \| - \| 25 (1 RCT) \| ⨁◯◯◯ MOLTO BASSA^a,b^ \|  \| \| Funzionamento adattivo \| La media funzionamento adattivo eran-a \| SMD 0.49 inferiore (1.2 inferiore a 0.22 maggiore) \| - \| 59 (2 RCT) \| ⨁◯◯◯ MOLTO BASSA^a,c,d^ \|  \| \| Interazione sociale \| La media interazione sociale eran-a \| SMD 0.27 maggiore (0.03 inferiore a 0.57 maggiore) \| - \| 172 (4 RCT) \| ⨁◯◯◯ MOLTO BASSA^a,e^ \|  \| \| Interessi e comportamenti ristretti e ripetitivi \| La media interessi e comportamenti ristretti e ripetitivi eran-a \| SMD 0.01 maggiore (0.36 inferiore a 0.39 maggiore) \| - \| 223 (6 RCT) \| ⨁⨁◯◯ BASSA^a^ \|  \| \| Comunicazione \| La media comunicazione era n-a SD \| SMD 0.05 SD inferiore (0.5 inferiore a 0.4 maggiore) \| - \| 223 (6 RCT) \| ⨁⨁◯◯ BASSA^a^ \|  \| \| Numero di eventi avversi \| Popolazione in studio \| \| **RR 1.54** (0.79 a 2.97) \| 157 (5 RCT) \| ⨁⨁◯◯ BASSA^f^ \|  \| \| 132 per 1,000 \| 203 per 1,000 (104 a 391) \|  1. Downgraded of two levels because population size <400 and there is a wide 95%CI, which includes no effect 2. Downgraded of one level because the measure used was the internalizing subscale of the BASC, which only indirectly measures anxiety 3. Downgraded of one level because one study is at high risk for incomplete outcome data and unclear risk for blinding and selective reporting 4. Downgraded of one level, because in one study the "social skills, parents assessed" of the subscale "adaptive skills" of the BASC was extracted 5. Downgraded of one level because in two studies Social interaction was analysed by the "inappropriate speech" subscale of the ABC, which relates more to behaviour and indirectly to social interaction 6. Downgraded of two levels because optimal information size (OIS) not met and there is a wide 95%CI, which includes no effect |  |
| Certezza delle prove Qual’è la certezza complessiva delle prove di efficacia e sicurezza? | | |
| Giudizi | Ricerca delle prove di evidenza | Considerazioni aggiuntive |
| ● Molto bassa ○ Bassa ○ Moderata ○ Alta ○ Nessuno studio incluso | La certezza delle prove è stata abbassata per imprecisione, rischio di distorsione sistematica, valutazioni indirette della misura di esito.  Complessivamente, la certezza delle prove è molto bassa (i due esiti critici "ansia" e "funzionamento adattivo" hanno una certezza delle prove molto bassa). |  |
| Valori C’è incertezza o variabilità su quanto le persone possano considerare importanti gli esiti principali? | | |
| Giudizi | Ricerca delle prove di evidenza | Considerazioni aggiuntive |
| ○ Importante incertezza o variabilità ● Probabilmente importante incertezza o variabilità  ○ Probabilmente non importante incertezza o variabilità  ○ Nessuna importante incertezza o variabilità | 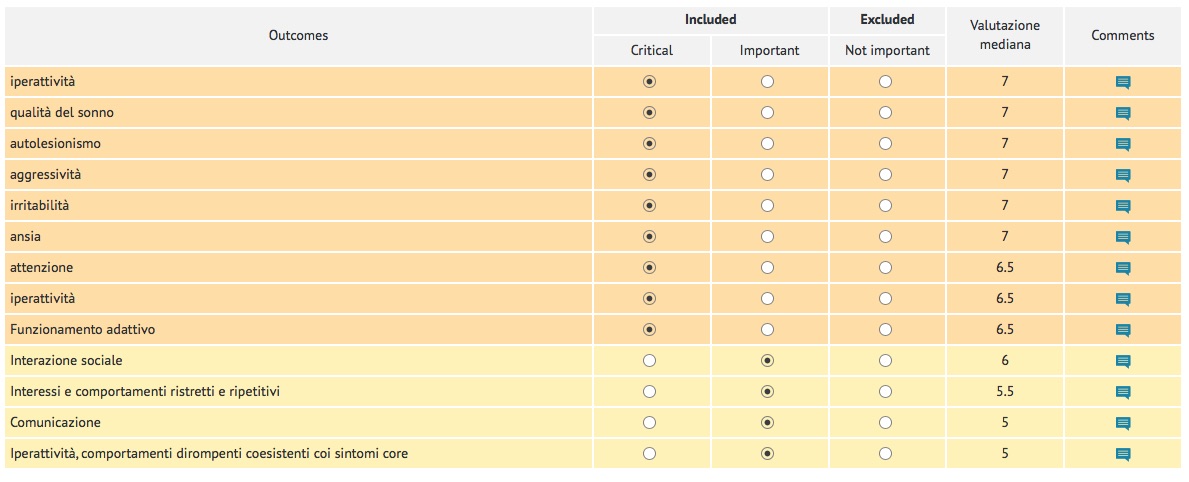  Recentemente, la Fondazione di pazienti americana "Autism Speaks" ha supportato gruppi di lavoro di esperti per valutare gli strumenti di misurazione di tre esiti importanti:  1) interessi ristretti e comportamenti ripetitivi (32);  2) Ansia (33);  3) Comportamenti di comunicazione sociale (34).  Non ci sono lavori sui Patient Reported Outcomes (PROMS) specifici per il disturbo dello spettro autistico (Digital Education Resource Archive, Oxford Patient-Reported Outcomes Measurement database), mentre è presente una revisione sistematica sulle misure di esito per bambini con disturbo dello spettro autistico, denominata progetto MeASURe (Measurement in Autism Spectrum disorder Under Review) (35). Il progetto MeASURe ha cercato di identificare valori specifici del bambino e / o della famiglia che i genitori dei bambini con ASD percepiscono come importanti, in tre modi diversi:  1) intraprendendo una revisione della letteratura qualitativa, usando le banche dati MEDLINE, CINAHL e PsycINFO; 2) conducendo una consultazione - tramite gruppi e via e-mail - per bambini ed adolescenti con disturbo dello spettro autistico; 3) facendo un sondaggio attraverso reti di professionisti della salute e dell'istruzione per esplorare quali esiti sono più spesso misurati dai professionisti della prima infanzia nel monitorare i progressi dei bambini.  Il progetto MeASUrE (35) ha riscontrato una notevole differenza tra gli esiti valutati importanti dai genitori e gli esiti più frequentemente misurati da clinici ed educatori. L'esperienza dei genitori con i loro figli li portano a enfatizzare risultati come il benessere emotivo dei bambini ed il loro funzionamento all'interno del nucleo famigliare. I clinici e gli educatori hanno riconosciuto di essere portati a misurare ciò per cui hanno gli strumenti. Inoltre, hanno riconosciuto che la loro pratica è influenzata da un'enfasi sulle caratteristiche core che definiscono lo spettro del disturbo autistico e sui disturbi del comportamento, quindi sono più portati a pensare come il bambino agisce sull'ambiente piuttosto che vedere il quadro più ampio e misurare come il bambino è influenzato dall' ambiente (35). Il progetto MeASUrE, dopo aver condotto interviste a clinici, a genitori ed a pazienti sui valori e le preferenze degli esiti, ha prodotto una lista di strumenti usati, raggruppati per dominio concettuale primario:    1) Severità dei sintomi autistici: Autism Behavior Checklist; Autism Diagnostic Interview-Revised (ADI-R); Autism Diagnostic Observation Schedule (ADOS, including Toddler Module and Calibrated Severity Score); Autism Observation Scale for Infants; The Baby and Infant Screen for Children with aUtIsm Traits-Part 1 (BISCUIT); Behavioral Summarized Evaluation (BSE-R; including Revised and Infant); Childhood Autism Rating Scale; Gilliam Autism Rating Scale (GARS and GARS-2); Modified Checklist for Autism in Toddlers; Parent Observation of Early Markers Scale; Pervasive Developmental Disorders Rating Scale; Social Communication Questionnaire; Social Responsiveness Scale (SRS).    2) Misure globali di esiti: Autism Treatment and Evaluation Checklist; Pervasive Developmental Disorders Behavior Inventory (PDDBI).    3) Coscienza sociale: Imitation Battery; Preschool Imitation and Praxis Scale (PIPS).    4) Interessi ristretti e ripetitivi: Repetitive Behavior Scale-Revised.    5) Sensory processing: Sense and Self-Regulation Checklist; Sensory Profile including Short Sensory Profile.    6) Linguaggio: MacArthur–Bates Communicative Development Inventories (MCDI); Preschool Language Scale-Fourth Edition.    7) Abilità cognitive: Leiter International Performance Scale-Revised; Mullen Scales of Early Learning; Stanford–Binet Intelligence Scales-Fifth Edition.    8) Regolazione emotiva: Baby and Infant Screen for Children with aUtIsm Traits-Part 2 (BISCUIT-Part 2); Children’s Global Assessment Scale; Infant–Toddler Social–Emotional Assessment (including Brief form).    9) Gioco: Test of Pretend Play.    10) Problemi comportamentali: Child Behavior Checklist (CBCL 1.5–5 and CBCL 6–18); Aberrant Behavior Checklist; BISCUIT-Part 3; Home Situations Questionnaire-Pervasive Developmental Disorders (HSQ-PDD) version; Nisonger Child Behavior Rating Form.    11) Misure globali di funzionamento: Global measure of functioning Behavior Assessment System for Children-Second Edition; Psychoeducational Profile-Revised (and Third Edition); Scales of Independent Behavior-Revised; Vineland Adaptive Behavior Scales (VABS; including Classroom and Screener versions).    12) Stress Genitoriale: Autism Parenting Stress Index; Parenting Stress Index-Short Form (PSI-SF); Questionnaire on Resources and Stress-Friedrich Short Form. |  |
| Bilanciamento degli effetti Il bilanciamento tra effetti desiderabili ed indesiderabili favorisce l’intervento o il confronto? | | |
| Giudizi | Ricerca delle prove di evidenza | Considerazioni aggiuntive |
| ○ E’ in favore del confronto ○ Probabilmente è in favore del confronto ●  Non è in favore né dell’intervento né del confronto ○ Probabilmente è in favore dell’intervento ○ E’ in favore dell’intervento ○ Varia ○ Non lo so |  |  |
| Risorse necessarie Quanto grandi sono le risorse necessarie (costi)? | | |
| Giudizi | Ricerca delle prove di evidenza | Considerazioni aggiuntive |
| ○ Costi molto elevati ●  Costi moderati  ○ Costi e risparmi irrilevanti ○ Risparmi moderati  ○ Grandi risparmi ○ Varia ○ Non so | **Il costo** **della supplementazione con PUFA nel mondo**  Uno studio Statunitense ha identificato nei supplementi il mezzo più economico, subito dopo l’olio di fegato di pesce, per assumere un dosaggio elevato (≥500 mg/die) di PUFA quali DHA ed EPA. Il costo dell’equivalente di una compressa contenente 1000 mg di EPA più DHA risultava infatti pari a 0.88$ ± 0.16$ (36). Analogamente, secondo un altro studio americano, che ha preso in considerazione un numero di supplementi presenti sul mercato più elevato, il costo dell’equivalente di una compressa contenente 1000 mg di EPA più DHA era di 0.70$ ± 1.11$ (37).    **Il costo della supplementazione con PUFA in Italia**  Prendendo in considerazione gli RCT inclusi per il calcolo dell’efficacia, si ottiene che la dose giornaliera mediana di PUFA somministrati per la terapia dei sintomi dell’autismo è pari a 1155 mg/die, e i dosaggi vanno da un minimo di 200 mg/die a un massimo di 1540 mg/die.  Il prezzo del farmaco in Italia invece è stato ricavato selezionando tutti i farmaci attualmente in commercio e prescrivibili in classe A per patologia secondo le note AIFA 13 e 94. I dati relativi al numero di capsule per confezione, il dosaggio della singola capsula e il prezzo della confezione sono stati ricavati da Farmadati Italia Srl (38). Dalle schede tecniche dei farmaci così selezionati si evince che su 1000 mg di prodotto si ritrovano all’incirca 850 mg di principio attivo. Dall’analisi di questi dati si ricava che il prezzo mediano per 1000 mg di prodotto netto è di 0.68 euro, e va da un minimo di 0.65 euro a un massimo di 0.83 euro.  Tenendo in considerazione i dati suddetti riguardanti le posologie utilizzate negli RCT e i prezzi dei farmaci, il costo di una giornata di terapia potrebbe dunque variare da 0.13 euro a 1.28 euro, con un valore mediano di 0.78 euro.  Negli RCT vengono effettuati dei cicli di trattamento di durata molto variabile, dalle 6 alle 52 settimane, con una mediana di 12 settimane. Considerando questa variabilità, assieme alla variabilità di prezzo e di dosaggio, si può calcolare che, in un presunto scenario tipico in cui venisse effettuato un ciclo di 3 mesi a una posologia di 1155 mg/die di PUFA al costo di 0.68 euro/1000mg, il costo di un ciclo di terapia sarebbe pari a 65.51 euro. L’analisi di sensitività completa, con gli scenari di spesa minimo e massimo, è presentata in tabella.  Non sono disponibili dati sul costo specifico per bambini ed adolescenti con disturbo dello spettro autistico. | Costi moderati- 11  Costi o risparmi trascurabili- 6  Astenuto-1 |
| Certezza delle prove relativamente alle risorse necessarie Qual’è la certezza delle prove relativamente alle risorse necessarie (costi)? | | |
| Giudizi | Ricerca delle prove di evidenza | Considerazioni aggiuntive |
| ● Molto bassa ○ Bassa ○ Moderata ○ Alta ○ Nessuno studio incluso | Ci sono incertezze riguardanti il costo di compresse con bassi dosaggi di EPA più DHA.  Infatti, tra i farmaci in classe A per la prevenzione secondaria delle malattie cardiovascolari, non vi era alcuna compressa di dosaggio inferiore a 500mg di PUFA, mentre per lo scenario di spesa minimo è stato preso in considerazione un dosaggio di 200mg/die, formulazione per la quale non erano disponibili dati di costo. I costi degli scenari di spesa minima potrebbero essere quindi leggermente superiori, visto il peso relativo dei costi fissi di confezionamento e distribuzione. |  |
| Costo efficacia L’analisi di costo efficacia favorisce l’intervento o il confronto? | | |
| Giudizi | Ricerca delle prove di evidenza | Considerazioni aggiuntive |
| ○ È in favore del confronto ○ Probabilmente è in favore del confronto ○ Non è in favore né del confronto né dell’intervento ○ Probabilmente è in favore dell’intervento ○ È in favore dell’intervento ○ Varia ● Nessuno studio incluso | Non ci sono dati di costo-efficacia in letteratura per l'utilizzo di Omega-3 in bambini ed adolescenti con disturbo dello spettro autistico. |  |
| Equità Quale sarebbe l’impatto in termini di equità? | | |
| Giudizi | Ricerca delle prove di evidenza | Considerazioni aggiuntive |
| ○ Riduce l’equità ● Probabilmente riduce l’equità ○ Probabilmente nessun impatto ○ Probabilmente migliora l’equità ○ Migliora l’equità ○ Varia ○ Non lo so | Il farmaco, non avendo come indicazione la terapia del disturbo dello spettro autistico (ASD), non è rimborsato dal servizio sanitario nazionale (SSN) ed è completamente a carico della famiglia del paziente. I costi di un ciclo di trattamento non sono ben definiti ma non dovrebbero superare i 466,54 euro/anno (vedi sezione Risorse richieste).  Secondo diversi studi, il titolo di studio dei genitori influenza la scelta di intraprendere una terapia alternativa o complementare, quale quella con PUFA (39), così come il carico percepito della terapia in termini di tempo, denaro ed energie influenzerebbe l’aderenza al trattamento. L’assunzione di farmaci o supplementi dovrebbe comunque avere un basso impatto sull’impegno da parte dei caregiver familiari, essendo un compito relativamente concreto e circoscritto per i genitori (40). |  |
| Accettabilità L’intervento è accettabile per i principali stakeholders? | | |
| Giudizi | Ricerca delle prove di evidenza | Considerazioni aggiuntive |
| ○ No ○ Probabilmente no ○ Probabilmente si ○ Si ● Varia ○ Non lo so | L’uso degli omega-3 negli individui affetti da disturbo dello spettro autistico sembra piuttosto diffuso: In uno studio osservazionale americano viene mostrato come la totalità dei bambini ed adolescenti con disturbo dello spettro autistico presi in considerazione effettuavano qualche forma di terapia, a cui il 55% affiancava l’utilizzo di almeno un supplemento nutrizionale; la supplementazione nutrizionale veniva considerata utile dal 50% dei genitori di individui con disturbo dello spettro autistico (ASD). Gli omega-3 erano utilizzati dal 18% dei pazienti considerati (42). Secondo altri studi, invece, a far uso di PUFA negli USA sarebbe il 51% dei bambini con ASD (43).  Secondo uno studio volto a valutare l’aderenza riportata dai genitori ai trattamenti per l’ASD, a seconda del tipo di trattamento seguito, l’aderenza media al trattamento con approccio alternativo, quale l’utilizzo di supplementi come gli omega-3 o la dieta sana, risultava essere significativamente inferiore rispetto all’aderenza alla terapia farmacologica o alla terapia evolutiva, mentre era sovrapponibile alla terapia comportamentale. Sempre secondo questo studio, un importante fattore predittivo di aderenza è risultato essere il peso percepito della terapia sulla famiglia in termini di tempo, energie, denaro (40). Un’altra possibile criticità nell’assunzione dei supplementi di PUFA da parte dei bambini con disturbo dello spettro autistico potrebbe essere dovuta alla difficoltà nel deglutire le capsule contenenti il medicinale (44).  Una possibile alternativa, utilizzata in alcuni studi, sarebbe quella di fornire i PUFA in una formulazione liquida, più adatta ai bambini, soprattutto se di età pre-scolare. Tuttavia, la stessa formulazione liquida, se accompagnata dalle caratteristiche sensoriali che tipicamente caratterizzano i supplementi contenenti olio di pesce, potrebbe potenzialmente ridurre la compliance al trattamento (44), (45), (23). In uno degli studi clinici randomizzati, per favorire l’aderenza al trattamento, si è deciso di optare per una capsula contenente un dosaggio inferiore di PUFA (200mg/die) e si afferma che, nonostante la supplementazione con dosaggi superiori sia stata presa in considerazione, questa è stata scartata proprio per le difficoltà nel somministrare anche solo una capsula al giorno nella popolazione autistica (18).  Riguardo l’accettabilità, quella dei PUFA non si discosta da quella del Placebo negli studi clinici, RR 1.01 (95%CI 0.66, 1.54) ed è stata studiata in 7 studi con una popolazione complessiva di 315 bambini ed adolescenti con disturbo dello spettro autistico (vedi tabella in basso). Studi osservazionali sembrano confermare la buona accettabilità dei PUFA (45) (25) (28) (27).  Diversi studi clinici randomizzati avevano inoltre tra gli obiettivi la valutazione dell’aderenza al trattamento, sia con metodiche soggettive che oggettive. In questi studi, l’aderenza è stata considerata da buona a eccellente (15), (12), (30), (18), (17), (23). Nel dettaglio, Mazahery riporta, nei bracci di pazienti randomizzati ad omega-3 e ad omega-3 più vitamina D, un aumento dell’indice di omega-3 rispettivamente del 4,4% e del 4% rispetto al baseline (14), mentre Voigt et al. (18) riportano un aumento significativo dei livelli di DHA circolante in tutti i soggetti randomizzati a PUFA, con un aumento mediano del 430% dei livelli di DHA plasmatico; Johnson et al (12) riportano che un solo partecipante allo studio (10%) non ha assunto regolarmente la medicazione, mentre nello studio di Bent et al (30), la compliance al trattamento era giudicata perfetta o quasi perfetta nel 69% dei pazienti randomizzati agli omega-3, contro il 75% dei pazienti randomizzati al placebo (75%). Percentuali molto elevate di aderenza (97%) sono riportate in entrambi i bracci da Parellada et al (17).  Infine, tra le evidenze riportate dagli studi osservazionali, una clinica psichiatrica prescolastica per il disturbo dello spettro autistico in Israele ha suggerito l'assunzione di Omega-3 a 250 bambini ed adolescenti con disturbo dello spettro autistico (41). Di questi, circa due terzi hanno accettato di prendere Omega-3. La metà di quelli che hanno accettato di prendere Omega-3 ha poi smesso l'assunzione perchè non ha visto alcun miglioramento o per il cattivo sapore. Non sono stati riscontrati altre barriere diverse dal cattivo sapore (41).     \| **Esiti** \| **Effetto assoluto anticipato^*^ (95% CI)** \| \| **Effetto relativo (95% CI)** \| **№ dei partecipanti (studi)** \| **Certainty of the evidence (GRADE)** \| **Commenti** \| \| --- \| --- \| --- \| --- \| --- \| --- \| --- \| \| **Rischio con placebo** \| **Rischio con acidi grassi poli-insaturi** \| \| Discontinuazione dovuta a qualsiasi causa \| Popolazione in studio \| \| **RR 1.01** (0.66 a 1.54) \| 315 (7 RCT) \| ⨁⨁◯◯ BASSA^a^ \|  \| \| 213 per 1.000 \| 215 per 1.000 (141 a 328) \|  1. Downgraded of two levels because optimal information size (OIS) not met and there is a wide 95%CI, which includes no effect |  |
| Fattibilità E’ fattibile l’implementazione dell’intervento? | | |
| Giudizi | Ricerca delle prove di evidenza | Considerazioni aggiuntive |
| ○ No ○ Probabilmente no ● Probabilmente si  ○ Si ○ Varia ○ Non lo so | Abbiamo effettuato una ricerca bibliografica per identificare barriere e facilitatori che dessero delle indicazioni sulla fattibilità del trattamento con PUFA in bambini ed adolescenti con disturbo dello spettro autistico. Al fine di trovare studi sulla fattibilità, abbiamo valutato le revisioni sistematiche e gli studi clinici provenienti dalla ricerca sull’efficacia e la sicurezza dei PUFA e le revisioni sistematiche e gli studi clinici provenienti dalla ricerca sui valori e preferenze.  Alcuni studi hanno mostrato come i medici non siano percepiti dai genitori di soggetti con disturbo dello spettro autistico (ASD) come sufficientemente ben informati riguardo le terapie alternative per l’ASD, tra le quali figura quella con i PUFA (42). La maggior parte dei genitori sceglierebbe di intraprendere una terapia alternativa o complementare da medici alternativi, infermieri e nutrizionisti. I medici dovrebbero essere in grado di intraprendere, con i genitori dei soggetti con ASD, una discussione sull’efficacia e i possibili rischi dei trattamenti alternativi o complementari (39), tra cui quello con PUFA.  L’assunzione dei supplementi di PUFA da parte del paziente autistico pediatrico presenta tra nelle possibili difficoltà nell’applicazione anche l’aderenza al trattamento, sia nel convincere il paziente ad assumere il supplemento (difficoltà nella deglutizione delle compressa, caratteristiche sensoriali sgradevoli del prodotto) (44), (45), (23) (18), sia per la propensione dei genitori a reputare utile o efficace il trattamento (40).  Tuttavia, negli studi considerati, l’aderenza alla terapia è generalmente buona o eccellente (14), (12), (30), (18), (17), (23), e così anche l’accettabilità, sia negli RCT inclusi che negli studi osservazionali (45) (25) (28) (27).    L’utilizzo di medicinali alternativi e di supplementi nutrizionali è in crescita nei bambini ed adolescenti con disturbo dello sviluppo. La crescita di questi agenti è alta soprattutto per quelle patologie nelle quali esiste incertezza circa il trattamento più efficace, o quando questo è gravato da importanti effetti collaterali. La crescita della prescrizione off-label e la vendita di molti PUFA come agenti nutraceutici, non come farmaci, ha aumentato l'accessibilità a questi prodotti. Tuttavia, il controllo sulla qualità nei prodotti nutraceutici è minore rispetto a quello sul farmaco, con potenziali rischi per la sicurezza (46). |  |

# Riassunto dei Giudizi

|  | **Giudizi** | | | | | | |
| --- | --- | --- | --- | --- | --- | --- | --- |
| **Problema** | No | Probabilmente no | Probabilmente si | Si |  | Varia | Non so |
| **Effetti desiderabili** | Irrilevanti | Piccoli | Moderati | Grandi |  | Varia | Non so |
| **Effetti Indesiderabili** | Grandi | Moderati | Piccoli | Irrilevanti |  | Varia | Non so |
| **Certezza delle Prove** | Molto bassa | Bassa | Moderata | Alta |  |  | Nessuno studio incluso |
| **Valori** | Importante incertezza o variabilità | Probabilmente importante incertezza o variabilità | Probabilmente nessuna importante incertezza o variabilità | Nessuna importante incertezza o variabilità |  |  |  |
| **Bilanciamento degli effetti** | A favore del confronto | Probabilmente a favore del confronto | Non è favorevole né al confronto né all’intervento | Probabilmente a favore dell’intervento | A favore dell’intervento | Varia | Non so |
| **Risorse richieste** | Costi elevati | Costi moderati | Costi e risparmi irrilevanti | Risparmi moderati | Grandi risparmi | Varia | Non so |
| **Certezza delle prove relativamente alle risorse necessarie** | Molto bassa | Bassa | Moderata | Alta |  |  | Nessuno studio incluso |
| **Costo efficacia** | A favore del confronto | Probabilmente a favore del confronto | Non è favorevole né al confronto né all’intervento | Probabilmente a favore dell’intervento | A favore dell’intervento | Varia | Nessuno studio incluso |

| **Equita’** | Riduce l’equità | Probabilmente riduce l’equità | Probabilmente nessun impatto sull’equità | Probabilmente aumenta l’equità | Aumenta l’equità | Varia | Non so |
| --- | --- | --- | --- | --- | --- | --- | --- |
| **Accettabilità** | No | Probabilmente no | Probabilmente si | Si |  | Varia | Non so |
| **Fattibilità** | No | Probabilmente no | Probabilmente si | Si |  | Varia | Non so |

# Tipo di raccomandazione

| Forte raccomandazione contro l’intervento | Raccomandazione condizionale contro l’intervento | Raccomandazione condizionale a favore sia dell’intervento che del confronto | Raccomandazione condizionale a favore dell’intervento | Forte raccomandazione a favore dell’intervento |
| --- | --- | --- | --- | --- |
| ○ | • | ○ | ○ | ○ |

# Conclusioni

| Raccomandazione |
| --- |
| Il gruppo della linea guida del ISS, suggerisce di non usare acidi grassi poli-insaturi in bambini e adolescenti con disturbi dello spettro autistico (raccomandazione condizionata, certezza della prove molto bassa). |
|  |

| Giustificazione |
| --- |
| Nessuna. |

| Considerazioni relative ai sottogruppi |
| --- |
| Nessuna. |

| Considerazioni per l’implementazione |
| --- |
| Nessuna. |

| Monitoraggio e valutazione |
| --- |
| Nessuna. |

| Priorità della ricerca |
| --- |
| Studi randomizzati controllati, con una definizione chiara degli esiti. |
